# Detection of Brain Tau Pathology in Down Syndrome Using Plasma Biomarkers

**DOI:** 10.1001/jamaneurol.2022.1740

**Published:** 2022-07-05

**Authors:** Shorena Janelidze, Bradley T. Christian, Julie Price, Charles Laymon, Nicole Schupf, William E. Klunk, Ira Lott, Wayne Silverman, H. Diana Rosas, Shahid Zaman, Mark Mapstone, Florence Lai, Beau M. Ances, Benjamin L. Handen, Oskar Hansson

**Affiliations:** 1 Clinical Memory Research Unit, Department of Clinical Sciences Malmö, Lund University, Lund, Sweden; 2 Waisman Center, University of Wisconsin, Madison; 3 Harvard Medical School, Department of Radiology, Massachusetts General Hospital, Charlestown; 4 Department of Psychiatry, University of Pittsburgh, Pittsburgh, Pennsylvania; 5 Taub Institute for Research on Alzheimer’s Disease and the Aging Brain, Columbia University Irving Medical Center, New York, New York; 6 School of Medicine, Department of Pediatrics, University of California, Irvine; 7 Harvard Medical School, Department of Neurology, Massachusetts General Hospital, Charlestown; 8 School of Clinical Medicine, Department of Psychiatry, University of Cambridge, Cambridge, United Kingdom; 9 Department of Neurology, University of California, Irvine; 10 Washington University School of Medicine in St Louis, St Louis, Missouri; 11 Memory Clinic, Skåne University Hospital, Malmö, Sweden

## Abstract

**Importance:**

Novel plasma biomarkers, especially phosphorylated tau (p-tau), can detect brain tau aggregates in Alzheimer disease.

**Objective:**

To determine which plasma biomarker combinations can accurately detect tau pathological brain changes in Down syndrome (DS).

**Design, Setting, and Participants:**

The cross-sectional, multicenter Alzheimer’s Biomarker Consortium–Down Syndrome study included adults with DS and a control group of siblings without DS. All participants with plasma, positron emission tomography (PET), and cognitive measures available by the time of data freeze 1.0 were included. Participants were enrolled between 2016 and 2019, and data were analyzed from August 2021 to April 2022.

**Exposures:**

Plasma p-tau217, glial fibrillary acidic protein (GFAP), amyloid β42/40 (Aβ42/Aβ40), neurofilament light (NfL), and total tau (t-tau); tau positron emission tomography (tau-PET) and Aβ-PET.

**Main Outcomes and Measures:**

The primary outcome was tau-PET status. Secondary outcomes included Aβ-PET status and cognitive performance.

**Results:**

Among 300 participants with DS and a control group of 37 non-DS siblings, mean (SD) age was 45.0 (10.1) years, and 167 (49.6%) were men. Among participants with DS who all underwent plasma p-tau217 and GFAP analyses, 258 had other plasma biomarker data available and 119, 213, and 288 participants had tau-PET, Aβ-PET, and cognitive assessments, respectively. Plasma p-tau217 and t-tau were significantly increased in Aβ-PET-positive tau-PET-positive (A^+^T^+^) DS and A^+^T^–^ DS compared with A^–^T^–^ DS while GFAP was only increased in A^+^T^+^ DS. Plasma p-tau217 levels were also significantly higher in A^+^T^+^ DS than A^+^T^–^ DS. In participants with DS, plasma p-tau217 and GFAP (but not other plasma biomarkers) were consistently associated with abnormal tau-PET and Aβ-PET status in models covaried for age (odds ratio range, 1.59 [95% CI, 1.05-2.40] to 2.32 [95% CI, 1.36-3.96]; *P* < .03). A combination of p-tau217 and age performed best when detecting tau-PET abnormality in temporal and neocortical regions (area under the curve [AUC] range, 0.96-0.99). The most parsimonious model for Aβ-PET status included p-tau217, t-tau, and age (AUC range, 0.93-0.95). In multivariable models, higher p-tau217 levels but not other biomarkers were associated with worse performance on DS Mental Status Examination (β, −0.24, 95% CI, −0.36 to −0.12; *P* < .001) and Cued Recall Test (β, −0.40; 95% CI, −0.53 to −0.26; *P* < .001).

**Conclusions and Relevance:**

Plasma p-tau217 is a very accurate blood-based biomarker of both tau and Aβ pathological brain changes in DS that could help guide screening and enrichment strategies for inclusion of individuals with DS in future AD clinical trials, especially when it is combined with age as a covariate.

## Key Points

**Question:**

How well do different plasma biomarker combinations detect Alzheimer disease–related brain tau pathology in Down syndrome?

**Findings:**

In this cross-sectional study including 300 participants with Down syndrome, plasma p-tau217 accurately distinguished individuals with abnormal tau positron emission tomography scans from those with normal scans, especially when combined with age.

**Meaning:**

Plasma p-tau217 is an accurate blood-based biomarker of tau pathological brain changes in Down syndrome that could be used as a stand-alone blood-based biomarker or in combinations with age to help guide screening and enrichment strategies for inclusion of people with Down syndrome in future Alzheimer disease clinical trials.

## Teaser

This cross-sectional study analyzes data from participants in the Alzheimer’s Biomarker Consortium–Down Syndrome study to determine which plasma biomarker combinations can accurately detect Alzheimer disease–related tau pathological brain changes in individuals with Down syndrome.

## Introduction

Down syndrome (DS) is associated with a variety of clinical manifestations resulting from additional copies of protein-encoding genes located on chromosome 21, including the gene for the amyloid precursor protein (APP) that is cleaved to produce amyloid β (Aβ).^1^ Overexpression of the *APP* gene in DS leads to accumulation of brain Aβ and tau pathologies typical of Alzheimer disease (AD).^2^ While more than 90% of people with DS develop AD pathological changes by the age of 40 years, the age at onset of cognitive symptoms ranges widely, from before 50 years to after 70 years.^3^ Therefore, biomarkers are needed to support clinical diagnosis and inclusion of people with DS without symptoms of dementia in AD clinical trials. Positron emission tomography (PET) imaging studies have revealed that increased regional binding of Aβ and tau radiotracers is similar in both DS and AD.^4,5,6^ Cerebrospinal fluid (CSF) biomarkers of AD-related brain Aβ pathology (Aβ42/40), tau pathology (phosphorylated tau [p-tau]), and neurodegeneration (neurofilament light [NfL]) have shown promise as diagnostic and prognostic biomarkers of AD in DS.^7,8^ However, for implementation in clinical practice and drug trials, inexpensive, noninvasive, scalable, and easily accessible blood biomarkers are needed.

Recent articles have demonstrated that plasma tau phosphorylated at threonine 181 (p-tau181) and NfL accurately differentiated adults with DS classified as asymptomatic from those with clinical diagnosis of prodromal AD or AD dementia.^9,10,11,12^ However, NfL is a nonspecific marker of neurodegeneration; its levels increase in normal aging, as well as in many disorders of the central nervous system (including DS), with no clinical evidence of AD.^10,13,14^ Plasma p-tau is a more specific biomarker of AD pathology, showing high concordance with tau-PET in patients with sporadic AD but not in other tauopathies.^13,15,16,17^ Because AD-related tau pathology is tightly linked to cognitive decline, tau-PET is increasingly used in clinical trials of sporadic AD to select appropriate study populations and to monitor treatment response.^13,18^ Considering that the costs of PET are high, it is likely that future clinical trials will include initial screening with plasma biomarkers to enroll individuals who are more likely to have brain tau pathology and rule out those who do not need to undergo additional procedures. However, relations between tau-PET imaging and plasma biomarkers (including plasma p-tau) in DS are at present unknown, and understanding these relations is important to facilitate future inclusion of individuals with DS in clinical trials of AD.

The overall aim of this study was to determine optimal combinations of plasma biomarkers to detect AD-related pathology in DS. To this end, we measured plasma concentrations of p-tau217, NfL, Aβ42/Aβ40, and total tau (t-tau) in individuals with DS. We selected plasma p-tau217 because previous findings in sporadic AD have suggested that CSF and plasma levels of p-tau217 might more accurately reflect AD pathology than p-tau181.^17,19,20,21^ We further measured plasma glial fibrillary acidic protein (GFAP), a marker of astrogliosis, which like p-tau217 has not been previously studied in DS. Plasma GFAP is increased in response to abnormal brain Aβ accumulation very early in the AD continuum and partly mediates the association between Aβ-PET and tau-PET.^22,23,24^ Our primary outcome was positivity on tau-PET, and we also investigated associations of plasma biomarkers with Aβ-PET measures and cognitive function. We tested whether combining p-tau217 and GFAP together and with other plasma AD biomarkers (Aβ42/40, NfL, t-tau) and age (a strong predictor of AD pathological changes in DS^25^) could further improve their discriminative accuracy for tau-PET and Aβ-PET status.

## Methods

The Alzheimer’s Biomarker Consortium–Down Syndrome study (ABC-DS)^26^ is conducted under institutional review board–approved protocols with participants and/or caregivers providing written informed consent to participate. The study followed the Strengthening the Reporting of Observational Studies in Epidemiology (STROBE) reporting guideline for cross-sectional studies.

### Participants

In this study, we included 300 participants with DS and 37 siblings without DS (control group) with baseline blood samples who were enrolled in the ABC-DS between July 13, 2016, and January 15, 2019, at multiple enrolling sites.^27^ Participants with DS received a diagnosis of cognitively stable (DS-CS, n = 212), mild cognitive impairment (DS-MCI, n = 40), or Alzheimer disease dementia (DS-dementia, n = 33) or were classified as “unable to determine” (n = 15). Cognitive function was evaluated with the Down Syndrome Mental Status Examination (DS-MSE)^28^ and Cued Recall Test (CRT),^29^ 2 measures included in a larger neuropsychological battery (full battery described in Handen et al^27^). Additional details regarding diagnostic procedures and cognitive testing are included in the eMethods in the Supplement.

### Plasma Sampling and Analysis

Plasma p-tau217 concentration was measured according to the published protocols using immunoassay on a Mesoscale Discovery platform developed by Lilly Research Laboratories.^17,30^ Briefly, biotinylated-IBA493 was used as a capture antibody and SULFO-TAG-4G10-E2 (anti-Tau) as the detector, and samples were diluted 1:2. The assay was calibrated with a synthetic p-tau217 peptide. Plasma GFAP concentration was quantified using a Simoa kit (Quanterix) according to the manufacturer’s instructions. Plasma Aβ42/Aβ40, NfL, and t-tau were analyzed with a Simoa 4-plex kit (Quanterix) in plasma samples from 258 participants with DS and 28 non-DS siblings as previously described.^12^ Plasma sampling and analysis are further described in the eMethods in the Supplement. All samples were analyzed by staff who were blinded to the clinical and imaging data.

### Tau- and Aβ-PET Imaging and Processing

Of 337 participants, 233 (213 with DS and 30 non-DS siblings) had carbon 11–labeled Pittsburgh compound B (PiB) PET scans, and 154 (119 DS and 35 non-DS siblings) underwent fluorine 18–labeled AV-1451 PET imaging. Tau-PET and Aβ-PET procedures are described in the eMethods in the Supplement. Previously described thresholds were used to define positivity for tau-PET (AV-1451 standardized uptake value ratio [SUVR] ≥1.3)^20^ and Aβ-PET ([^11^C]PiB centiloid ≥33.3).^31,32^ We also performed a sensitivity analysis using a lower Aβ-PET threshold of centiloid 18 that was derived for predicting future Aβ accumulation in participants with DS.^33^

### Statistical Analysis

Data were analyzed from August 2021 to April 2022. SPSS version 27 (IBM) and R version 4.0.3 (RStudio version 1.3)^34^ were used for statistical analysis. Group differences in log-transformed plasma biomarker levels were assessed using analysis of covariance (with Bonferroni correction for multiple comparisons) adjusting for age and sex. Associations of log-transformed plasma biomarkers with continuous Aβ-PET centiloid, tau-PET SUVR, or cognitive test scores were examined with linear regression models that included age and sex and additionally the level of premorbid intellectual impairment (cognition only) as covariates. Untransformed data are presented in figures to aid interpretation of biomarker values across different comparisons. Associations between plasma biomarkers and Aβ-PET or tau-PET status were tested using logistic regression models and receiver operating characteristic curve (ROC) analysis. Plasma biomarkers were *z* scored according to mean and SD values in the Aβ-PET negative non-DS sibling group to make results more comparable. We first tested whether individual biomarkers were associated with abnormal PET status independent of age. Biomarkers with *P* values less than .10 were included in subsequent analysis to find the most parsimonious models. For that, we used the Multi-Model-Inference R package that tests all combinations of variables and ranks the models according to the Akaike information criterion (AIC). Performance of models was considered similar if ΔAIC was less than 2. Area under the curve (AUC) of 2 ROCs were compared using the DeLong test.

## Results

### Participants

Of 337 participants, 167 (49.6%) were men, and the mean (SD) age was 45.0 (10.1) years (Table 1). In participants with DS in the ABC-DS cohorts, Aβ-PET starts to become abnormal at approximately 35 years of age (eFigure 1 in the Supplement). Therefore, we performed a sensitivity analysis including only people with DS who were 35 years or older (eTable 1 in the Supplement), because including very young individuals with DS where all were Aβ-PET negative might inflate the diagnostic accuracy of the biomarkers. The numbers of DS participants with plasma biomarker, PET, and cognitive measures available by the time of data freeze 1.0 who were included in different analyses are shown in eFigure 2 in the Supplement.

**Table 1.** Demographic and Clinical Characteristics

	Median (IQR) or No.	
	DS (n = 300)	Non-DS siblings (n = 37)
Age, y	46.0 (37.3-53.0)	45.0 (32.0-54.0)
Sex, No.		
Female	139	31
Male	161	6
Diagnosis, No.		
Cognitively stable	212	NA
Mild cognitive impairment	40	NA
Alzheimer disease dementia	33	NA
Not determined	15	NA
DS-MSE	62.5 (50.8-72.0)	NA
No.	289	NA
CRT	33.0 (22.0-35.0)	NA
No.	272	NA
Premorbid intellectual impairment, No.		
Mild	156	NA
Moderate	113	NA
Severe	30	NA
Plasma p-tau217, pg/mL	0.541 (0.381-0.946)	0.329 (0.278-0.409)
Plasma GFAP, pg/mL	166.8 (95.9-305.5)	88.3 (56.6-116.2)
Plasma Aβ42/Aβ40	0.034 (0.031-0.036)	0.036 (0.032-0.038)
No.	258	28
Plasma t-tau, pg/mL	2.40 (1.89-3.10)	1.82 (1.56-2.40)
No.	258	28
Plasma NfL, pg/mL	15.6 (10.0-27.0)	7.23 (4.90-8.70)
No.	258	28
Aβ-PET, PiB centiloid	22.2 (3.18-61.2)	−0.8 (−3.3-2.1)
No.	213	30
Aβ-PET positivity, No. (%)	85 (39.9)	1 (2.7)
Tau-PET, [^18^F]AV-1451 SUVR, temporal meta-ROI	1.12 (1.07-1.17)	1.12 (1.09-1.15)
No.	119^a^	35^a^
Tau-PET positivity, temporal meta-ROI, No. (%)	14 (11.8)	0
Tau-PET, [^18^F]AV-1451 SUVR, neocortical meta-ROI	1.05 (0.99-1.11)	1.07 (1.03-1.09)
No.	119^a^	35^a^
Tau-PET positivity, neocortical meta-ROI, No. (%)	10 (8.4)	0

Abbreviations: Aβ, β-amyloid; CRT, Cued Recall Test; DS-MSE, Down Syndrome Mental Status Examination; GFAP, glial fibrillary acidic protein; NA, not available; NfL, neurofilament light chain; PET, positron emission tomography; PiB, Pittsburgh compound B; p-tau, phosphorylated tau; ROI, region of interest; SUVR, standardized uptake value ratio; t-tau, total tau.

^a^ Of 154 participants with tau-PET, 139 participants (111 with DS and 28 non-DS siblings) also underwent Aβ-PET.

### Plasma Biomarkers by Diagnostic Groups and PET Status

Plasma concentrations of p-tau217, GFAP, and NfL were already increased in participants with DS-CS compared with non-DS siblings and further increased in those with DS-MCI and DS-dementia (eFigure 3 in the Supplement). There were no significant differences in plasma Aβ42/Aβ40, whereas t-tau increased with the onset of cognitive symptoms (eFigure 3 in the Supplement).

**Figure 1.**
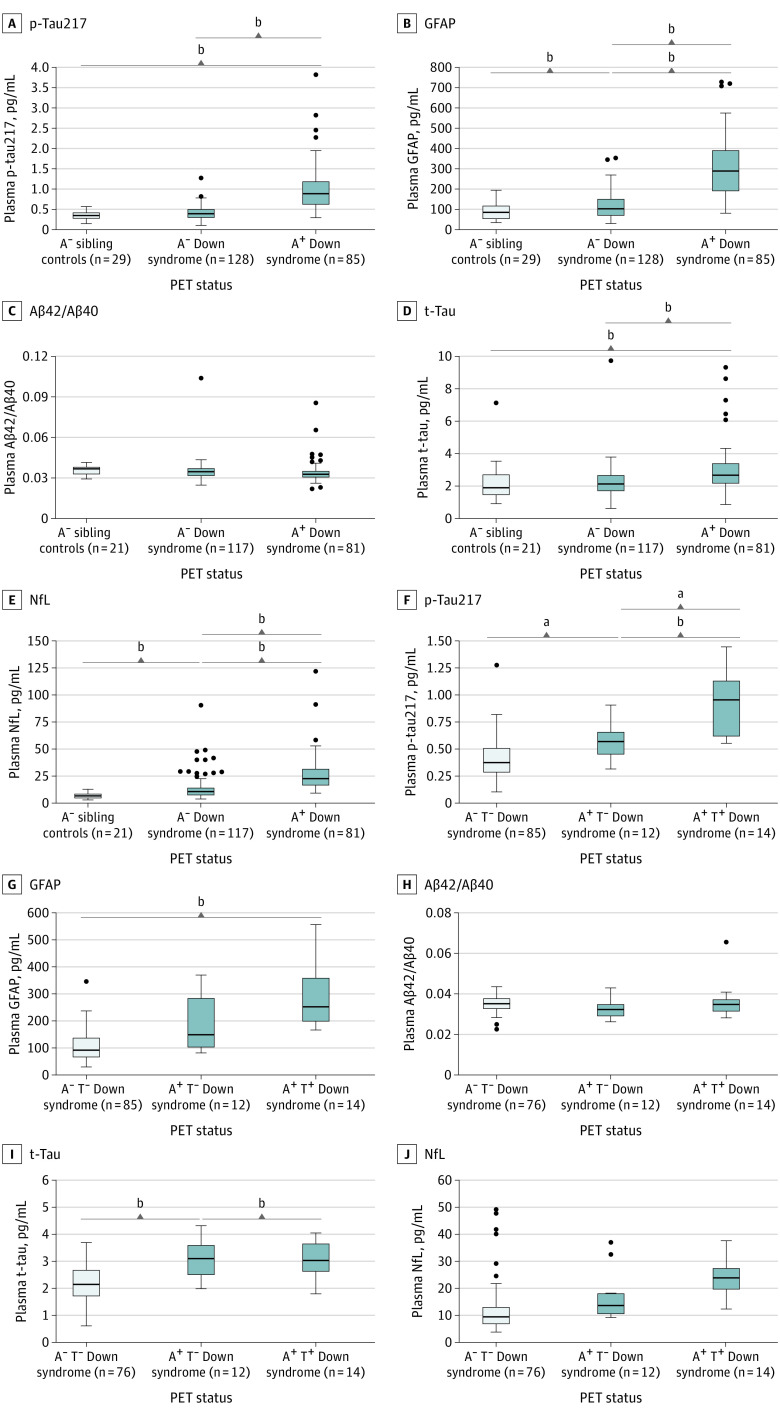
Plasma Biomarkers by Positron Emission Tomography (PET) Status for Participants With Down Syndrome (DS) and a Control Group of Siblings Without DS Plasma concentrations of the biomarkers were compared between Aβ-PET negative (A^–^) non-DS siblings, A^–^ DS and A^+^ DS, and between A^–^ participants with DS who were tau-PET negative in the temporal meta region of interest (A^–^ T^–^), A^+^ T^–^ DS, and A^+^ T^+^ DS. *P* values are from univariate general linear models adjusted for age and sex with Bonferroni correction for multiple comparisons. Boxes show IQR, horizontal lines are medians, and whiskers and outliers were plotted using the Tukey method. Aβ indicates amyloid β; GFAP, glial fibrillary acidic protein; NfL, neurofilament light chain; p-tau, phosphorylated tau; t-tau, total tau. ^a^*P* < .05. ^b^*P* < .001.

When diagnostic groups were stratified by Aβ-PET (A^+^ or A^–^) status (Figure 1A-E), we found that p-tau217 levels were higher in A^+^ DS compared with both A^–^ DS (mean difference [MD], 0.33; 95% CI, 0.26 to 0.41; *P* < .001) and A^–^ non-DS siblings (MD, 0.40; 95% CI, 0.30 to 0.50; *P* < .001), but there were no differences between A^–^ DS and A^–^ non-DS siblings (MD, 0.07; 95% CI, −0.03 to 0.16; *P* = .29). Interestingly, GFAP concentration was increased in A^–^ DS compared with A^–^ non-DS siblings (MD, 0.20; 95% CI, 0.10 to 0.29; *P* < .001) and even more increased in A^+^ DS (MD, 0.44; 95% CI, 0.34 to 0.54; *P* < .001). Similar to p-tau217, t-tau levels were higher in A^+^ DS than in the 2 A^–^ groups (MD, 0.18; 95% CI, 0.10 to 0.26, and MD, 0.18; 95% CI, 0.06 to 0.29; *P* < .001) with no differences between A^–^ DS and A^–^ non-DS siblings. NfL, like GFAP, was increased in both the A^–^ DS (MD, 0.29; 95% CI, 0.17 to 0.41; *P* < .001) and A^+^ DS groups (MD, 0.44; 95% CI, 0.31 to 0.56; *P* < .001) compared with A^–^ non-DS siblings. Plasma Aβ42/Aβ40 did not differ between the groups.

When further stratifying by tau-PET (T^+^ or T^–^) status (Figure 1F-J), we observed that plasma levels of p-tau217, but not other plasma biomarkers, were higher in both A^+^T^–^ DS (MD, 0.16; 95% CI, 0.02-0.30; *P* = .02) and A^+^T^+^ DS (MD, 0.35; 95% CI, 0.19-0.50; *P* < .001) than in A^–^T^–^ DS and were also higher in A^+^T^+^ DS compared with A^+^T^-^ DS (MD, 0.19; 95% CI, 0.01-0.36; *P* = .03). Of note, none of the T^+^ participants were A^–^. While there were no differences in plasma GFAP or t-tau between A^+^T^-^ DS and A^+^T^+^ DS, the biomarkers levels were lower in A^–^T^–^ DS than in the other groups (GFAP: A^–^T^–^ DS vs A^+^T^+^ DS; MD, 0.27; 95% CI, 0.11-0.43; *P* < .001; t-tau: A^–^T^–^ DS vs A^+^T^+^ DS; MD, 0.23; 95% CI, 0.08-0.38; *P* < .001, and A^–^T^–^ DS vs A^+^T^–^ DS; MD, 0.23; 95% CI, 0.09-0.36; *P* < .001). Aβ42/Aβ40 and NfL did not differ between any of the groups.

### Associations With Tau-PET and Aβ-PET

**Figure 2.**
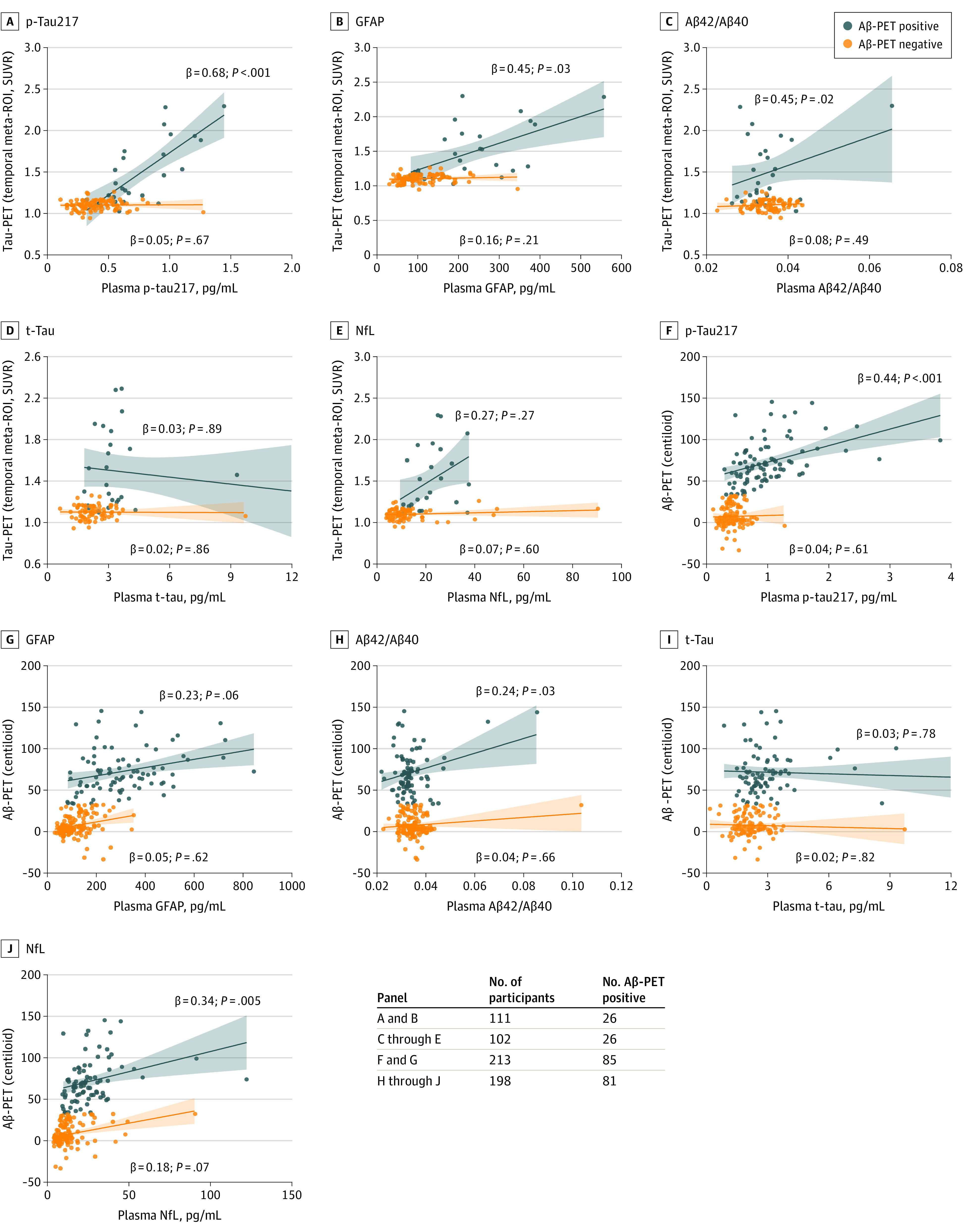
Associations Between Plasma Biomarkers and Tau Positron Emission Tomography (PET) and Amyloid β (Aβ) PET Measures in Participants With Down Syndrome (DS) Associations of plasma biomarkers with tau-PET SUVR in the temporal meta-region of interest (ROI) and Aβ-PET centiloid. Data are shown as β (standardized coefficient) and *P* value from linear regression models adjusted for age and sex. Two total tau (t-tau) values not shown in panels D and I were included in the statistical analysis. Aβ indicates amyloid β; GFAP, glial fibrillary acidic protein; NfL, neurofilament light chain; p-tau, phosphorylated tau; SUVR, standardized uptake value ratio.

Higher levels of plasma p-tau217 and GFAP correlated with increased tau-PET SUVR in the temporal region (p-tau217: standardized β = 0.68; 95% CI, 0.31 to 1.04; *P* < .001; GFAP: β = 0.45; 95% CI, 0.05 to 0.85; *P* = .03) and Aβ-PET centiloid (p-tau217: β = 0.44; 95% CI, 0.24 to 0.63; *P* < .001; GFAP: β = 0.23; 95% CI, −0.01 to 0.46; *P* = .06) in A^+^ DS but not in A^–^ DS (Figure 2). We found no associations between either NfL or t-tau with tau-PET measures (Figure 2). Plasma NfL was positively associated with Aβ-PET in A^+^ DS (β = 0.34; 95% CI, 0.10 to 0.57; *P* = .005). Higher plasma Aβ42/Aβ40 was associated with increased tau-PET and Aβ-PET signal in A^+^ DS (Figure 2), but these associations were no longer significant after excluding 2 outliers (data not shown).

We next studied how well different plasma biomarkers or their combinations could detect abnormality on tau-PET or Aβ-PET in participants with DS. Age is a strong predictor of AD pathological changes in DS^25^; therefore, we included age in the analysis. Both tau-PET and plasma biomarker measures were available in 109 participants with DS (eFigure 2A in the Supplement), of whom 14 (12.8%) had abnormal tau-PET signal in the temporal region. Univariable analysis showed that p-tau217, GFAP, NfL, and age were significantly associated with abnormal tau-PET (Table 2). However, the associations were independent of age only for p-tau217 (OR, 2.02; 95% CI, 1.32-3.11; *P* = .001) and GFAP (OR, 1.73; 95% CI, 1.14-2.62; *P* = .01). The best-performing model among the top 4 models was the most parsimonious one, including only 2 predictors, p-tau217 and age (AUC, 0.96; 95% CI, 0.92-1.0) (Table 3 and eFigure 4A in the Supplement).

**Table 2.** Associations With Tau-PET and Aβ-PET in Participants With Down Syndrome^a^

	Model performance					
	OR (95% CI)	*P* value	AUC (95% CI)	AIC	SN	SP
**Tau-PET temporal meta-ROI**						
Univariable models						
Age	1.32 (1.15-1.51)	<.001	0.92 (0.85-0.98)	53.4	1.00	0.71
p-Tau217	2.13 (1.52-2.99)	<.001	0.94 (0.88-0.99)	50.3	1.00	0.77
GFAP	2.08 (1.49-2.89)	<.001	0.94 (0.90-0.98)	55.6	1.00	0.84
NfL	1.29 (1.06-1.57)	.01	0.88 (0.81-0.95)	80.1	0.93	0.81
t-Tau	1.24 (0.93-1.65)	.15	0.77 (0.64-0.89)	85.6	0.79	0.74
Aβ42/Aβ40	1.15 (0.85-1.56)	.36	0.51 (0.35-0.67)	86.8	0.50	0.74
Multivariable models (age + plasma biomarker)						
p-Tau217	2.02 (1.32-3.11)	.001	0.96 (0.92-1.0)	36.5	0.93	0.88
GFAP	1.73 (1.14-2.62)	.01	0.95 (0.92-0.99)	46.7	1.00	0.87
t-Tau	1.51 (1.00-2.27)	.05	0.93 (0.87-0.99)	52.7	0.93	0.81
Aβ42/Aβ40	1.34 (0.93-1.93)	.11	0.93 (0.88-0.99)	52.9	0.93	0.79
NfL	1.02 (0.83-1.27)	.83	0.92 (0.86-0.98)	55.4	1.00	0.73
**Aβ-PET**						
Univariable models						
Age	1.23 (1.16-1.30)	<.001	0.87 (0.82-0.92)	180.7	0.95	0.64
p-Tau217	2.24 (1.76-2.85)	<.001	0.91 (0.87-0.95)	149.7	0.75	0.93
GFAP	2.01 (1.69-2.59)	<.001	0.89 (0.84-0.94)	162.7	0.79	0.86
NfL	1.57 (1.34-1.85)	<.001	0.84 (0.78-0.90)	226.2	0.82	0.81
t-Tau	2.57 (1.56-4.24)	<.001	0.71 (0.64-0.78)	249.1	0.70	0.62
Aβ42/Aβ40	0.95 (0.83-1.09)	.44	0.61 (0.53-0.69)	271.2	0.73	0.53
Multivariable models (age + plasma biomarker)						
p-Tau217	2.08 (1.58-2.73)	<.001	0.94 (0.91-0.97)	119.6	0.82	0.95
GFAP	1.69 (1.36-2.11)	<.001	0.91 (0.87-0.95)	149.5	0.72	0.92
t-Tau	2.58 (1.49-4.48)	<.001	0.90 (0.86-0.94)	158.9	0.79	0.85
NfL	1.17 (1.00-1.37)	.05	0.87 (0.83-0.92)	178.2	0.90	0.72
Aβ42/Aβ40	0.93 (0.80-1.07)	.29	0.87 (0.82-0.92)	181.5	0.90	0.69

Abbreviations: Aβ, β-amyloid; AIC, Akaike information criteria; AUC, area under the curve; GFAP, glial fibrillary acidic protein; NfL, neurofilament light chain; OR, odds ratio; PET, positron emission tomography; p-tau, phosphorylated tau; ROC, receiver operating characteristic curve; ROI, region of interest; SN, sensitivity; SP, specificity; t-tau, total tau.

^a^ Data are from logistic regression models and ROC analysis with tau-PET status as outcome. For plasma biomarkers, ORs represent increased risk of PET positivity for each SD change in biomarker value.

**Table 3.** Model Selection and Performance for Detecting Abnormal Tau-PET and Aβ-PET in Participants With Down Syndrome^a^

	Model				Age		p-Tau217		GFAP		t-Tau		NfL	
	AUC (95% CI)	AIC	SN	SP	OR (95% CI)	*P* value	OR (95% CI)	*P* value	OR (95% CI)	*P* value	OR (95% CI)	*P* value	OR (95% CI)	*P* value
**Tau-PET temporal meta-ROI**														
Age, p-tau217^b^	0.96 (0.92-1.0)	36.5	0.93	0.88	1.31 (1.09-1.56)	.004	2.02 (1.32-3.11)	.001						
Age, p-tau217, GFAP	0.97 (0.94-1.0)	37.2	0.93	0.91	1.25 (1.03-1.52)	.03	1.89 (1.20-2.97)	.006	1.30 (0.81-2.07)	.28				
Age, p-tau217, t-tau	0.96 (0.92-1.0)	38.4	0.93	0.87	1.30 (1.08-1.56)	.005	1.99 (1.27-3.10)	.002			1.12 (0.46-2.75)	.81		
Age, p-tau217, GFAP, t-tau	0.97 (0.94-1.0)	39.2	0.93	0.91	1.25 (1.02-1.53)	.03	1.89 (1.17-3.05)	.009	1.30 (0.81-2.08)	.28	1.00 (0.42-2.38)	.99		
**Aβ-PET**														
Age, p-tau217, GFAP, t-tau	0.96 (0.93-0.98)	107.1	0.83	0.97	1.17 (1.07-1.28)	<.001	1.94 (1.45-2.61)	<.001	1.35 (1.01-1.79)	.04	1.93 (1.13-3.30)	.02		
Age, p-tau217, GFAP, t-tau, NfL	0.96 (0.93-0.98)	107.7	0.81	0.98	1.19 (1.08-1.31)	<.001	1.97 (1.46-2.64)	<.001	1.39 (1.04-1.87)	.03	1.98 (1.13-3.44)	.02	0.88 (0.69-1.11)	.28
Age, p-tau217, t-tau^b^	0.95 (0.93-0.98)	109.7	0.89	0.89	1.23 (1.13-1.33)	<.001	2.10 (1.57-2.81)	<.001			1.96 (1.12-3.42)	.02		
Age, p-tau217, t-tau, NfL	0.96 (0.93-0.98)	111.0	0.83	0.97	1.24 (1.14-1.36)	<.001	2.12 (1.58-2.85)	<.001			2.00 (1.12-3.56)	.02	0.92 (0.74-1.14)	.43

Abbreviations: Aβ, β-amyloid; AIC, Akaike information criteria; AUC, area under the curve; GFAP, glial fibrillary acidic protein; NfL, neurofilament light chain; OR, odds ratio; PET, positron emission tomography; p-tau, phosphorylated tau; ROC, receiver operating characteristic curve; ROI, region of interest; SN, sensitivity; SP, specificity; t-tau, total tau.

^a^ Data are from logistic regression models and ROC analysis with tau-PET and Aβ-PET status as outcomes. Models are ordered based on AIC (lower values representing better model fit), and performance of models was considered similar if a difference in AIC was <2. The OR and *P* value for the variable included in each model are reported. For plasma biomarkers, ORs represent increased risk of PET positivity for each SD change in biomarker value.

^b^ Parsimonious model.

The results were very similar when using tau-PET SUVR in the neocortical meta–region of interest (ROI) as the outcome (eResults and eFigures 4 and 5 in the Supplement). While p-tau217, GFAP, NfL, and age were significantly associated with abnormal tau-PET, the associations were independent of age only for p-tau217 (OR, 2.32; 95% CI, 1.36-3.96; *P* = .002) and GFAP (OR, 1.59; 95% CI, 1.05-2.40; *P* = .03) (eTable 2 in the Supplement). The best-performing model included p-tau217 and age (AUC, 0.99; 95% CI, 0.96-1.00) (eTable 3 and eFigure 4B in the Supplement).

Aβ-PET and plasma biomarker data were all available in 198 participants with DS (81 [40.9%] Aβ-PET positive) (eFigure 2A in the Supplement). In univariable models, age and all plasma biomarkers except Aβ42/Aβ40 were significantly associated with abnormal Aβ-PET status (Table 2). The associations were independent of age for p-tau217 (OR, 2.08; 95% CI, 1.58-2.73; *P* < .001), GFAP (OR, 1.69; 95% CI, 1.36-2.11; *P* < .001), and t-tau (OR, 2.58; 95% CI, 1.49-4.48; *P* < .001). When testing different combinations of plasma biomarkers and age, we found the top model that fit the data best included p-tau217, GFAP, t-tau, and age (AUC, 0.96; 95% CI, 0.93-0.98) (Table 3 and eFigure 4C in the Supplement). However, a more parsimonious model (ie, a model with fewer predictors) combining p-tau217, t-tau, and age performed equally well in terms of both AUC (0.95; 95% CI, 0.93-0.98; *P* = .44 for difference) and AIC (ΔAIC, −2.6) (Table 3 and eFigure 4C in the Supplement). Sensitivity analysis using a centiloid 18 cutoff to define Aβ status did not alter the overall findings. Although p-tau217 (OR, 1.45; 95% CI, 1.17-1.80; *P* < .001), GFAP (OR, 1.60; 95% CI, 1.23-2.09; *P* < .001), t-tau (OR, 1.69; 95% CI, 1.11-2.57; *P* = .01), and NfL (OR, 1.36; 95% CI, 1.08-1.71; *P* = .009) were all significantly associated with abnormal Aβ-PET when adjusting for age, the model including p-tau217, t-tau, and age (AUC, 0.93; 95% CI, 0.90-0.97) performed as well as the best model (ΔAUC, −0.01; ΔAIC, −2.8) (eTable 4 and 5 in the Supplement). As expected, the AUCs for individual biomarkers were somewhat lower with this lower threshold for Aβ positivity.

### Associations With Cognition

Increased levels of plasma p-tau217 (β, −0.31; 95% CI, −0.40 to −0.22; and β, −0.50; 95% CI, −0.60 to −0.40; *P* < .001) and GFAP (β, −0.28; 95% CI, −0.40 to −0.16; and β, −0.42; 95% CI, −0.56 to −0.23; *P* < .001) were associated with lower DS-MSE and CRT scores (eTable 6 in the Supplement). We also found significant associations of plasma t-tau and NfL with the DS-MSE and CRT measures (range: β, −0.11; 95% CI, −0.20 to −0.02; to β, −0.37; 95% CI, −0.51 to −0.23; *P* < .03) (eTable 6 in the Supplement). However, in the models combining the 4 plasma biomarkers (eTable 6 in the Supplement), only p-tau217 was independently associated with DS-MSE (β, −0.24; 95% CI, −0.36 to −0.12; *P* < .001) and CRT (β, −0.40; 95% CI, −0.53 to −0.26; *P* < .001). When stratified by Aβ-PET status, increased levels of plasma p-tau217 were associated with worse performance on cognitive tests (DS-MSE: β, −0.23; 95% CI, −0.40 to −0.07; *P* = .007; and CRT: β, −0.56; 95% CI, −0.74 to −0.37; *P* < .001) in A^+^ DS but not A^–^ DS (eFigure 6 in the Supplement).

### Sensitivity Analysis in Participants With DS Who Were 35 Years and Older

The results were very similar when younger participants with DS were included in the analysis (eTable 1 and 7-9 and eFigure 2B in the Supplement). A combination of p-tau217 and age showed high discriminative accuracy for abnormal vs normal tau-PET in temporal (AUC, 0.96; 95% CI, 0.90-1.0) and neocortical (AUC = 0.98; 95% CI, 0.96-1.0) regions. The model including p-tau217, t-tau, and age had an AUC of 0.95 (95% CI, 0.91-0.98) when using Aβ-PET status as the outcome. Full details of the sensitivity analysis are presented in the eResults in the Supplement.

## Discussion

In this study, we show, for the first time to our knowledge, that in participants with DS, p-tau217 and GFAP but not other plasma AD biomarkers (ie, Aβ42/Aβ40, t-tau, and NfL) were associated with tau-PET status when accounting for age. At the same time, among all tested biomarker combinations, a composite measure of p-tau217 and age showed the highest accuracy (AUC >0.95) for distinguishing participants with DS and abnormal tau-PET scans from those with normal tau-PET scans. For detection of Aβ-PET status, the most parsimonious model included p-tau217, age, and t-tau (AUC >0.93). In addition, we report that plasma p-tau217 and t-tau were increased in A^+^ DS but not in A^–^ DS, compared with A^–^ non-DS siblings, whereas GFAP and NfL were increased in both A^+^ DS and A^–^ DS groups. Furthermore, while plasma p-tau217, GFAP, and t-tau were all increased in A^+^T^+^ DS compared with A^–^T^–^ DS, p-tau217 was the only biomarker with higher levels in A^+^T^+^ DS than A^+^T^–^ DS. Higher levels of p-tau 217 were consistently associated with worse performance on cognitive tests.

Plasma p-tau is currently considered the most promising biomarker of brain tau and Aβ pathologies in sporadic and autosomal dominant AD.^13^ Although the discriminative accuracy of plasma p-tau181 for Aβ-PET status in DS has recently been reported,^11^ associations between plasma biomarkers and tau-PET have not been studied in this population, and it is also unclear if combinations of different plasma biomarkers offer improved accuracy for identifying individuals with DS who have abnormal tau-PET or Aβ-PET scans. Here we found that increased levels of plasma p-tau217, GFAP, t-tau, and NfL were all associated with abnormal tau-PET status in the temporal region and that age was associated with tau-PET positivity. However, our results also indicated that the associations with tau-PET were independent of age for only p-tau217 and GFAP and not the other plasma biomarkers. Furthermore, the strongest correlations with tau-PET SUVR in A^+^ DS was seen for plasma p-tau217. The best-performing model to identify DS participants with abnormal tau-PET signal included only p-tau217 and age and showed very high discriminative accuracy (AUC >0.95) with no added value of other plasma biomarkers.

Similar analysis for Aβ-PET as an outcome revealed that plasma p-tau217, GFAP, and t-tau were associated with Aβ-PET status independent of age. The performance of p-tau217 by itself (AUC = 0.91) was considerably better than the previously reported performance of p-tau181 (AUC = 0.77).^11^ Although this is in keeping with findings in sporadic AD,^17,20,21^ we cannot rule out that differences in cohort characteristics and preanalytical sample handling might have affected the results. Thus, head-to-head comparison of different p-tau isoforms in the same cohorts of participants with DS are warranted, but it is unlikely that another plasma p-tau variant will greatly outperform the current p-tau217 assay because it performed very well in DS, with AUCs above 0.9 for both tau- and Aβ-PET. The most parsimonious model for Aβ-PET status included p-tau217, t-tau, and age differentiating DS with abnormal scans from DS with normal Aβ-PET scans with AUC of 0.93 to 0.95. The usefulness of plasma t-tau as a biomarker of Aβ pathology has not been observed in sporadic or autosomal dominant AD and is one of the novel findings of this study that should be replicated in future investigations.

Our results of increased plasma GFAP and NfL in A^–^ DS suggest that changes in these biomarker levels might be associated with very early dysregulation of Aβ metabolism occurring prior to formation of Aβ plaques visible on PET imaging. Nevertheless, it is also possible that plasma GFAP and NfL are affected by non-AD mechanisms and neurodevelopmental defects. NfL is a marker of neurodegeneration released from injured axons,^13,14^ so changes in NfL concentrations could be associated with the reduced regional brain volume and decreased neuronal numbers that characterize DS.^35^ In addition, immune dysfunction is common in DS because several immunoregulatory genes are positioned on chromosome 21. Activation of astrocytes caused by elevated levels of proinflammatory cytokines in the DS brain^36^ and increased astrocytic expression of S100B (a protein encoded by the gene on chromosome 21) could provide an explanation for increased plasma levels of GFAP in the present study and previously reported high CSF levels of another astrocytic marker, YKL-40, in asymptomatic DS.^7^

For people in the early stages of sporadic AD, elevated levels of plasma p-tau217, p-tau181, and NfL are associated with cognitive decline over time and conversion to AD dementia,^15,16,37,38^ whereas in DS, baseline and longitudinal changes in plasma NfL have been linked to the progression of cognitive symptoms.^9,39^ Our study demonstrated that at a cross-sectional level, high plasma p-tau217 concentration was independently associated with worse cognitive performance in participants with DS. We did not find significant associations between plasma NfL and cognitive scores when controlling for the association of p-tau217 with cognition, possibly because of the cross-sectional nature of the present study.

### Limitations

Lack of longitudinal data is one of the limitations of the present study. Future investigations in longitudinal samples are needed to explore association between plasma biomarkers and clinical progression. Another limitation is that the cohort size was relatively small, especially when considering the number of tau-PET–positive cases. However, there are currently no other larger DS cohorts worldwide with both tau-PET and Aβ-PET imaging data. In addition, we analyzed plasma Aβ42/Aβ40 using Simoa immunoassay and in sporadic AD, where mass spectrometry–based assays have shown clearly better performance than immunoassays when detecting brain Aβ pathology.^40^ Nevertheless, given the very high accuracy of p-tau217 observed in this study, it is unlikely that plasma Aβ42/Aβ40 could offer clinically meaningful improvement even if measured with mass spectrometry–based assays.

## Conclusions

Our study shows that in DS, plasma p-tau217 accurately identifies individuals with abnormal tau-PET and Aβ-PET scans (especially when combined with age). Because of overexpression of the *APP* gene, adults with DS are at very high risk of developing AD and consequently are likely to benefit from anti-amyloid or anti-tau therapies. However, patient selection into trials evaluating anti-amyloid or anti-tau drugs rely on CSF analysis and PET imaging procedures that are expensive, invasive, and not widely available. This has hindered the inclusion of people with DS from participation in such trials.^8,41^ The excellent performance of plasma p-tau217 in the present study indicates that it could be used even as a stand-alone blood-based biomarker enabling selection and inclusion of adults with DS in anti-AD clinical trials.

## Notes

Supplement.
eMethods

eResults
**eTable 1.** Demographic and clinical characteristics of study participants aged ≥35y **eTable 2.** Associations with tau-PET SUVR in the neocortical meta-ROI in participants with DS **eTable 3.** Model selection and performance for detecting abnormal tau-PET in the neocortical meta-ROI in participants with DS **eTable 4.** Associations with Aβ-PET in participants with DS using centiloid ≥18 for Aβ positivity **eTable 5.** Model selection and performance for detecting abnormal Aβ-PET in participants with DS using centiloid ≥18 cutoff for Aβ positivity **eTable 6.** Associations with cognition in participants with DS **eTable 7.** Associations with tau-PET in participants with DS aged ≥35y **eTable 8.** Associations with Aβ-PET in participants with DS aged ≥35y **eTable 9.** Associations with cognition in participants with DS aged ≥35 **eFigure 1.** Aβ-PET centiloid values as a function of age in participants with DS **eFigure 2.** The numbers of DS participants with plasma biomarker, PET and cognitive measures available included in the main (all participants, A) and sensitivity analyses (aged ≥35 years, B) **eFigure 3.** Plasma biomarker concentrations across diagnostic groups **eFigure 4.** ROC curve analyses for predicting abnormal Tau-PET status and Aβ-PET in participant with DS **eFigure 5.** Associations between plasma biomarkers and tau-PET SUVR in the neocortical region in participant with DS **eFigure 6.** Associations of plasma biomarkers with cognition in participant with DS
eReferences
**eTable 10.** Members of the Alzheimer’s Biomarker Consortium–Down Syndrome (ABC-DS)
