# Supplementary material for: Detection of Brain Tau Pathology in Down Syndrome Using Plasma Biomarkers
Source: JAMA Neurol. 2022 Jul 5;79(8):797–807. doi: 10.1001/jamaneurol.2022.1740 (PMC9257682; doi:10.1001/jamaneurol.2022.1740)
Supplement: Supplement. — eMethods eResults eTable 1. Demographic and clinical characteristics of study participants aged ≥35y eTable 2. Associations with tau-PET SUVR in the neocortical meta-ROI in participants with DS eTable 3. Model selection and performance for detecting abnormal tau-PET in the neocortical meta-ROI in participants with DS eTable 4. Associations with Aβ-PET in participants with DS using centiloid ≥18 for Aβ positivity eTable 5. Model selection and performance for detecting abnormal Aβ-PET in participants with DS using centiloid ≥18 cutoff for Aβ positivity eTable 6. Associations with cognition in participants with DS eTable 7. Associations with tau-PET in participants with DS aged ≥35y eTable 8. Associations with Aβ-PET in participants with DS aged ≥35y eTable 9. Associations with cognition in participants with DS aged ≥35 eFigure 1. Aβ-PET centiloid values as a function of age in participants with DS eFigure 2. The numbers of DS participants with plasma biomarker, PET and cognitive measures available included in the main (all participants, A) and sensitivity analyses (aged ≥35 years, B) eFigure 3. Plasma biomarker concentrations across diagnostic groups eFigure 4. ROC curve analyses for predicting abnormal Tau-PET status and Aβ-PET in participant with DS eFigure 5. Associations between plasma biomarkers and tau-PET SUVR in the neocortical region in participant with DS eFigure 6. Associations of plasma biomarkers with cognition in participant with DS eReferences eTable 10. Members of the Alzheimer’s Biomarker Consortium–Down Syndrome (ABC-DS) [file jamaneurol-e221740-s001.pdf]

Supplemental Online Content

Janelidze S, Christian BT, Price J, et al. Detection of brain tau pathology in Down syndrome using plasma biomarkers. *JAMA Neurol*. Published online July 5, 2022. doi:10.1001/jamaneurol.2022.1740

eMethods
eResults
eTable 1. Demographic and clinical characteristics of study participants aged ≥35y
eTable 2. Associations with tau-PET SUVR in the neocortical meta-ROI in participants with DS
eTable 3. Model selection and performance for detecting abnormal tau-PET in the neocortical meta-ROI in participants with DS
eTable 4. Associations with Aβ-PET in participants with DS using centiloid ≥18 for Aβ positivity
eTable 5. Model selection and performance for detecting abnormal Aβ-PET in participants with DS using centiloid ≥18 cutoff for Aβ positivity
eTable 6. Associations with cognition in participants with DS
eTable 7. Associations with tau-PET in participants with DS aged ≥35y
eTable 8. Associations with Aβ-PET in participants with DS aged ≥35y
eTable 9. Associations with cognition in participants with DS aged ≥35
eFigure 1. Aβ-PET centiloid values as a function of age in participants with DS
eFigure 2. The numbers of DS participants with plasma biomarker, PET and cognitive measures available included in the main (all participants, A) and sensitivity analyses (aged ≥35 years, B)
eFigure 3. Plasma biomarker concentrations across diagnostic groups
eFigure 4. ROC curve analyses for predicting abnormal Tau-PET status and Aβ-PET in participant with DS
eFigure 5. Associations between plasma biomarkers and tau-PET SUVR in the neocortical region in participant with DS
eFigure 6. Associations of plasma biomarkers with cognition in participant with DS
eReferences
eTable 10. Members of the Alzheimer’s Biomarker Consortium–Down Syndrome (ABC-DS)

This supplemental material has been provided by the authors to give readers additional information about their work.

© 2022 Janelidze S et al. *JAMA Neurology.*

eMethods

Participants

In this study, we included 300 participants with DS and 37 non-DS sibling controls with baseline blood samples who were enrolled in the Alzheimer's Biomarker Consortium- Down Syndrome study (ABC-DS; https://www.nia.nih.gov/research/abc-ds) between Jul 13, 2016, and Jan 15, 2019 at multiple enrolling sites.[1] ABC-DS is conducted under IRB approved protocols with participants and/or caregivers providing written informed consent to participate.

Participants with DS received a diagnosis of cognitively stable (DS-CS, n=212), mild cognitive impairment (DS-MCI, n=40), Alzheimer's disease dementia (DS-dementia, n=33) or were classified as "unable to determine" (n=15). Diagnosis was determined by clinical consensus conferences generally in accordance with the recommendations of the AAMR-IASSID Working Group for the Establishment of Criteria for the Diagnosis of Dementia in Individuals with Developmental Disability and was based on medical, clinical, and cognitive testing considered in reference to baseline IQ and any recent major life transitions or events. Neither genetic findings (e.g., APOE status) nor biomarker results were available to the consensus team members. Participants were classified as cognitively stable if they were without cognitive or functional decline, beyond what would be expected with adult aging, per se. Participants were classified as having MCI if they demonstrated some cognitive and/or functional decline over and above what would be expected with aging per se, but not severe enough to indicate the presence of dementia. Participants were categorized as having Alzheimer's disease dementia if there was evidence of substantial progressive declines in cognitive functioning and daily living skills. An "unable to determine" category was utilized to indicate that declines were observed but could be caused by significant life circumstance (e.g., staff changes, family death) or conditions unrelated to AD (e.g., severe sensory loss, poorly resolved hip fracture, psychiatric diagnosis primarily depression).

Cognitive function was evaluated with the Down Syndrome Mental Status Examination (DS-MSE)[2] and Cued Recall Test,[3] two measures included in a larger neuropsychological battery (see Handen et al.,[1] for full battery). These measures were selected because they are designed for use with individuals varying widely in their premorbid levels of intellectual functioning and have been shown to be sensitive to early signs of prodromal AD in adults with DS.[4-6] DS participants classified as "unable to determine" were included in the analysis examining associations of plasma biomarkers with Aβ-PET, tau-PET and cognition.

Plasma sampling and analysis

Plasma P-tau217 concentration was measured according to the published protocols using immunoassay on a Mesoscale Discovery platform developed by Lilly Research Laboratories.[7,8] Briefly, biotinylated-IBA493 was used as a capture antibody and SULFO-TAG-4G10-E2 (anti-Tau) as the detector and samples were diluted 1:2. The assay was calibrated with a synthetic P-tau217 peptide. Plasma GFAP concentration was quantified using Simoa kit (Quanterix, Lexington, MA, USA) according to the manufacturer's instructions. P-tau217 and GFAP were analyzed in plasma samples from 300 participants with DS and 37 non-DS sibling controls at the Clinical Memory Research Unit, Lund University (Sweden); one case with insufficient plasma volume for both P-tau217 and GFAP assays was excluded from the study. All measurements were above the lower limit of detection of the assay (P-tau217, 0.10 pg/ml; GFAP, 0.26 pg/ml). The inter-assay coefficient of variation (CV) for 3-4 quality control samples included in every run were 14.2% and 17.4% in the P-tau217 and GFAP assays, respectively. The intra-assay CV was 5.7% in the P-tau217 assay and 4.0% in the GFAP assay.

Plasma Aβ42/Aβ40, NfL and T-tau were analyzed with Simoa 4-plex kit (Quanterix, Lexington, MA, USA) in plasma samples from 258 participants with DS and 28 non-DS sibling controls at the Institute for Translational Research, University of North Texas Health Science Center (Fort Worth, USA) as previously described.[9]

All samples were analyzed by staff blinded to the clinical and imaging data.

Tau and Aβ PET imaging and processing

Out of 337 participants, 233 (213 DS and 30 non-DS sibling controls) had [^{11}C]Pittsburgh Compound-B (PiB) PET scans and 154 (119 DS and 35 non-DS sibling controls) underwent [^{18}F]AV-1451 PET imaging. PET images were acquired on a Siemens ECAT HR + (University of Wisconsin- Madison, University of Pittsburgh), Siemens 4-ring Biograph mCT (University of Pittsburgh), GE SIGNA (University of Cambridge), and GE Discovery 710 (Barrow Neurological Institute) scanners as previously described.10,11 Aβ-PET imaging was performed 50–70 min after the injection of 15 mCi (target dose) of PiB (four 5-minute frames). Following Aβ-PET, tau-PET was performed 80–100 min after the injection of 10 mCi (target dose) of [^{18}F]AV-1451 (four 5-minute frames). PET frames were inspected for motion, and if necessary, re-aligned using PMOD software. Frames were averaged to form a single 50-70 minute [^{11}C]PiB image and a single 80-100 minute [^{18}F]AV-1451 image for each subject. PET images were rigidly registered to their corresponding T1-weighted magnetic resonance (MR) images.

In the AV-1451 analysis, the T1 MR images were segmented into a standard set of regions of interest (ROIs) using FreeSurfer 5.3 (FS) that were transferred to the registered PET. A specific uptake value ratio (SUVR) for each FS ROI

was calculated by normalizing regional activity-concentration to cerebellar gray matter activity-concentration. Final tau measures were determined for meta-ROIs formed from groups of FS ROIs, including a temporal meta-ROI (entorhinal cortex, inferior and middle temporal cortices, fusiform gyrus, parahippocampal cortex and amygdala, approximating Braak I/ III/IV) and neocortical meta-ROI (caudal anterior cingulate gyrus, caudal middle frontal gyrus, cuneus, inferior parietal lobule, isthmus cingulate, lateral occipital gyrus, lateral orbitofrontal gyrus, lingual gyrus, medial orbitofrontal gyrus, paracentral gyrus, pars opercularis, pars orbitalis, pars triangularis, pericalcarine cortex, postcentral gyrus, posterior cingulate gyrus, precentral gyrus, precuneus, rostral anterior cingulate gyrus, rostral middle frontal gyrus, superior frontal gyrus, superior parietal lobule, superior temporal gyrus, supramarginal gyrus, frontal pole, temporal pole, transverse temporal lobe, insula, approximating Braak V/VI to capture late-stage tau pathology).[12,13] An SUVR for each meta-ROI was determined as a volume weighted average of the constituent FS-ROI SUVRs.

PiB PET was quantified in terms of a global centiloid value using established methodology.[14] Briefly, with the T1 MR image as an intermediary, [^{11}C]PiB images were spatially warped to the Montreal Neurological Institute 152 space (MNI152) using SPM8. A global SUVR was determined for each subject from tracer concentration in a standard cortex ROI (including anterior cingulate, frontal cortex, parietal cortex, precuneus, temporal cortex, and striatum) normalized to the concentration in a whole cerebellum ROI. Both ROIs are available at the GAAIN website (http://www.gaain.org/centiloid-project).

eResults

Associations with Tau-PET SUVR in the neocortical region

In Aβ-PET positive participants with DS (A+ DS), the association with tau-PET SUVR in the neocortical region was significant for P-tau217 (β=0.715, p<0.001, eFigure 5A) but not for GFAP (β=0.386, p=0.09, eFigure 4B). There were no significant associations between either of the two plasma biomarkers and tau-PET SUVR in the neocortical meta-ROI in A^{-} DS. We found no associations between either NfL or T-tau with tau-PET measures (eFigures 5D-E). Higher plasma Aβ42/Aβ40 was associated with increased tau-PET signal in A+ DS (eFigure 5C), but these associations were no longer significant after excluding two outliers (data not shown). Both tau-PET and plasma biomarker measures were available in 109 participants with DS (eFigure 2A) of whom 10 (9.2%) had abnormal tau-PET signal in the neocortical region. Univariable analysis showed that P-tau217, GFAP, NfL and age were significantly associated with abnormal tau-PET and that the associations were independent of age for both P-tau217 (OR=2.32, p=0.002)) and GFAP (OR=1.59, p=0.028) (eTable 2). The best performing model among the top 4 models was the most parsimonious one including only two predictors, i.e., P-tau217 and age (AUC=0.985, CI [0.962-1.00]) (eTable 3, eFigure 4B).

Sensitivity analysis in participants with DS aged ≥35y

The results were very similar when younger participants with DS were excluded from the analysis (eTable 2, eFigure 2B). Association with both tau-PET and Aβ-PET status were consistently significant for plasma P-tau217 and GFAP (eTables 7-8). A combination of P-tau217 and age showed high discriminative accuracy for abnormal vs normal tau-PET in both temporal (AUC=0.957, CI [0.903-1.0]) and neocortical (AUC=0.983, CI [0.957-1.0]) regions. The model including P-tau217, T-tau and age had an AUC of 0.947 (CI 0.912-0.981) when using Aβ-PET status as the outcome. In multivariable models, increased levels of plasma P-tau217 but not other plasma biomarkers, were associated with lower DS-MSE scores (β=-0.271, p<0.001) whereas associations with Cued Recall test scores were significant for both P-tau217 (β=-0.457, p<0.001) and T-tau (β=-0.116, p=0.048) (eTable 9).

© 2022 Janelidze S et al. *JAMA Neurology.*

eTable 1. Demographic and clinical characteristics of study participants aged ≥35y

	DS<br>n=253	Non-DS Sibling Controls<br>n=26
Age, *years*	48.0 (41.0-53.0)	49.5 (41.3-59.5)
Sex F/M, *n*	115/138	23/3
Diagnosis,<br>CS/MCI/ADD/not determined, n	166/40/33/14	NA
DS-MSE	60.0 (47.0-70.5)<br>N=244	NA
CRT	31.0 (20.0-35.0)<br>N=228	NA
Premorbid Intellectual Impairment, mild/moderate/severe, n	132/95/26	NA
Plasma P-tau217, *pg/ml*	0.575 (0.412-0.996)	0.322 (0.277-0.389)
Plasma GFAP, *pg/ml*	199.6 (115.9-339.4)	96.4 (63.8-122.8)
Plasma Aβ42/Aβ40	0.033 (0.031-0.036)<br>N=219	0.035 (0.031-0.037)<br>N=18
Plasma T-tau, *pg/ml*	2.45 (1.92-3.13)<br>N=219	1.75 (1.50-2.01)<br>N=18
Plasma NfL, *pg/ml*	18.6 (11.6-29.2)<br>N=219	8.45 (7.25-11.05)<br>N=18
Aβ-PET, PiB centiloid	32.3 (6.8-68.8)<br>N=171	-0.51 (-3.17-2.12)<br>N=21
Aβ-PET positivity, n (%)	84 (49.1%)	0 (0)
Tau-PET,<br>[^{18}F]AV-1451 SUVR, temporal meta-ROI	1.13 (1.08-1.19)<br>N=83*	1.12 (1.10-1.15)<br>N=24*
Tau-PET positivity, temporal meta-ROI, *n (%)*	14 (16.9%)	0 (0%)
Tau-PET,<br>[^{18}F]AV-1451 SUVR, neocortical meta-ROI	1.05 (0.99-1.11)<br>N=83*	1.07 (1.03-1.08)<br>N=24*
Tau-PET positivity, neocortical meta-ROI, *n (%)*	10 (12.0%)	0 (0%)

Data are shown median (interquartile range) unless otherwise specified.
* Out of 107 participants with tau-PET, 94 (77 DS and 21 non-DS sibling controls) also underwent Aβ-PET.

Abbreviations: Aβ=β-amyloid; ADD=AD dementia; CRT=Cued Recall test; CS=cognitively stable; DS-MSE=Down Syndrome Mental Status Examination; F=female; GFAP=glial fibrillary acidic protein; M=male; MCI=mild cognitive impairment; NA=not available; NfL=neurofilament light chain; PET=positron emission tomography; PiB= Pittsburgh compound B; P-tau=phosphorylated tau; ROI=region of interest; SUVR=standardized uptake value ratio; T-tau=total tau.

© 2022 Janelidze S et al. *JAMA Neurology.*

eTable 2. Associations with tau-PET SUVR in the neocortical meta-ROI in participants with DS

	**MODEL PERFORMANCE**				
**TAU-PET NEOCORTICAL META-ROI**					
**Univariable models**	**OR (p value)**	**AUC (95% CI)**	**AIC**	**SN**	**SP**
Age	1.34 (0.001)	0.921 (0.856-0.987)	44.0	1.00	0.74
P-tau217	2.35 (<0.001)	0.967 (0.930-1.000)	34.5	1.00	0.87
GFAP	1.95 (<0.001)	0.930 (0.877-0.984)	46.1	1.00	0.81
NfL	1.24 (0.031)	0.865 (0.789-0.942)	66.0	0.80	0.89
T-tau	1.16 (0.35)	0.824 (0.734-0.915)	70.1	0.90	0.73
Aβ42/Aβ40	1.24 (0.19)	0.514 (0.310-0.718)	69.2	0.40	0.72
**Multivariable models (age + plasma biomarker)**					
P-tau217	2.32 (0.002)	0.985 (0.962-1.000)	25.1	1.00	0.90
GFAP	1.59 (0.028)	0.945 (0.894-0.997)	39.8	1.00	0.84
Aβ42/Aβ40	1.52 (0.049)	0.942 (0.886-0.999)	41.9	0.80	0.95
T-tau	1.23 (0.51)	0.927 (0.868-0.987)	45.6	1.00	0.80
NfL	1.00 (1.00)	0.920 (0.852-0.988)	46.0	1.00	0.75

Data are from logistic regression models and ROC curve analysis with tau-PET status as outcome. For plasma biomarkers, odds ratios represent increased risk of tau-PET positivity for each SD change in biomarker value.

Abbreviations: Aβ=β-amyloid; AIC=Akaike information criteria; AUC=area under the curve; CI=confidence interval; GFAP=glial fibrillary acidic protein; NfL=neurofilament light chain; OR=odds ratio; PET=positron emission tomography; P-tau=phosphorylated tau; ROC= receiver operating characteristic curve; ROI=region of interest; SN=sensitivity; SP=specificity; T-tau=total tau.

© 2022 Janelidze S et al. *JAMA Neurology.*

eTable 3. Model selection and performance for detecting abnormal tau-PET in the neocortical meta-ROI in participants with DS

	Model				Odds ratio (p-value)		
**TAU-PET NEOCORTICAL META-ROI**	**AUC (95% CI)**	**AIC**	**SN**	**SP**	**Age**	**P-tau217**	**GFAP**
Age, P-tau217	0.985 (0.962-1.0)	25.1	1.00	0.90	1.34 (0.015)	2.32 (0.002)	
Age, P-tau217, GFAP	0.984 (0.961-1.0)	27.1	1.00	0.90	1.32 (0.050)	2.26 (0.005)	1.00 (0.81)
P-tau217, GFAP	0.974 (0.941-1.0)	30.9	1.00	0.87		2.03 (<0.001)	1.01 (0.034)
P-tau217	0.967 (0.930-1.0)	34.5	1.00	0.87		2.35 (<0.001)	

Data are from logistic regression models and ROC curve analysis with tau-PET as outcome. Models are ordered based on AIC (lower values representing better model fit). Odds ratio (p-value) of the variable included in each model are reported. For plasma biomarkers, odds ratios represent increased risk of tau-PET positivity for each SD change in biomarker value. The parsimonious models are highlighted.

Abbreviations: AIC=Akaike information criteria; AUC=area under the curve; CI=confidence interval; GFAP=glial fibrillary acidic protein; NfL=neurofilament light chain; OR=odds ratio; PET=positron emission tomography; P-tau=phosphorylated tau; ROC= receiver operating characteristic curve; ROI=region of interest; SN=sensitivity; SP=specificity; T-tau=total tau.

eTable 4. Associations with Aβ-PET in participants with DS using centiloid ≥18 for Aβ positivity

	MODEL PERFORMANCE				
**Aβ-PET**					
**Univariable models**	**OR (p value)**	**AUC (95% CI)**	**AIC**	**SN**	**SP**
Age	1.30 (<0.001)	0.905 (0.863-0.947)	155.5	0.90	0.79
GFAP	2.31 (<0.001)	0.881 (0.835-0.926)	175.2	0.76	0.85
NfL	2.15 (<0.001)	0.852 (0.798-0.905)	203.7	0.70	0.89
P-tau217	1.72 (<0.001)	0.821 (0.764-0.877)	204.5	0.60	0.90
T-tau	1.73 (0.012)	0.650 (0.573-0.726)	266.0	0.60	0.68
Aβ42/Aβ40	1.01 (0.77)	0.589 (0.509-0.669)	275.5	0.57	0.68
**Multivariable models (age + plasma biomarker)**					
P-tau217	1.45 (<0.001)	0.927 (0.893-0.962)	139.6	0.84	0.92
GFAP	1.60 (<0.001)	0.926 (0.892-0.961)	141.0	0.92	0.89
T-tau	1.69 (0.015)	0.920 (0.882-0.957)	147.3	0.77	0.94
NfL	1.36 (0.009)	0.918 (0.879-0.957)	149.0	0.86	0.86
Aβ42/Aβ40	1.04 (0.76)	0.905 (0.862-0.947)	157.3	0.89	0.79

Data are from logistic regression models and ROC curve analysis with tau-PET status as outcome. For plasma biomarkers, odds ratios represent increased risk of Aβ-PET positivity for each SD change in biomarker value.

Abbreviations: Aβ=β-amyloid; AIC=Akaike information criteria; AUC=area under the curve; CI=confidence interval; GFAP=glial fibrillary acidic protein; NfL=neurofilament light chain; OR=odds ratio; PET=positron emission tomography; P-tau=phosphorylated tau; ROC= receiver operating characteristic curve; ROI=region of interest; SN=sensitivity; SP=specificity; T-tau=total tau.

© 2022 Janelidze S et al. *JAMA Neurology.*

eTable 5. Model selection and performance for detecting abnormal Aβ-PET in participants with DS using centiloid ≥18 cutoff for Aβ positivity

	Model				Odds ratio (p-value)				
**Aβ-PET**	**AUC (95% CI)**	**AIC**	**SN**	**SP**	**Age**	**P-tau217**	**GFAP**	**T-tau**	**NfL**
Age, P-tau217, GFAP, T-tau	0.938 (0.907-0.969)	133.0	0.80	0.97	1.22 (<0.001)	1.32 (0.019)	1.39 (0.034)	1.43 (0.09)	
Age, P-tau217, GFAP, T-tau, NfL	0.940 (0.909-0.970)	133.5	0.86	0.92	1.21 (<0.001)	1.31 (0.026)	1.33 (0.070)	1.42 (0.08)	1.17 (0.24)
Age, P-tau217, T-tau, NfL	0.937 (0.905-0.968)	135.0	0.87	0.91	1.25 (<0.001)	1.40 (0.003)		1.47 (0.047)	1.24 (0.10)
Age, P-tau217, GFAP	0.934 (0.902-0.966)	135.4	0.81	0.94	1.12 (<0.001)	1.34 (0.011)	1.44 (0.016)		
Age, P-tau217, T-tau	0.933 (0.900-0.966)	135.8	0.84	0.93	1.27 (<0.001)	1.42 (0.002)		1.48 (0.047)	

Data are from logistic regression models and ROC curve analysis with tau-PET as outcome. Models are ordered based on AIC (lower values representing better model fit). Odds ratio (p-value) of the variable included in each model are reported. For plasma biomarkers, odds ratios represent increased risk of Aβ-PET positivity for each SD change in biomarker value. The parsimonious models are highlighted.

Abbreviations: AIC=Akaike information criteria; AUC=area under the curve; CI=confidence interval; GFAP=glial fibrillary acidic protein; NfL=neurofilament light chain; OR=odds ratio; PET=positron emission tomography; P-tau=phosphorylated tau; ROC= receiver operating characteristic curve; ROI=region of interest; SN=sensitivity; SP=specificity; T-tau=total tau.

eTable 6. Associations with cognition in participants with DS

Biomarker	DS-MSE	CRT
**Models including individual plasma biomarkers**	**β (p value), N**	**β (p value), N**
P-tau217	-0.307 (0.001), N=288	-0.497 (0.001), N=271
GFAP	-0.280 (0.001), N=288	-0.417 (0.001), N=271
T-tau	-0.107 (0.022), N=248	-0.239 (0.001), N=234
NfL	-0.142 (0.025), N=248	-0.368 (0.001), N=234
Aβ42/Aβ40	-0.022 (0.64), N=248	-0.053 (0.35), N=234
**Multivariable models combining plasma P-tau217, GFAP, NfL, T-tau***		
P-tau217	-0.238 (<0.001)	-0.398 (<0.001)
GFAP	-0.076 (0.33)	-0.050 (0.56)
T-tau	-0.045 (0.33)	-0.090 (0.087)
NfL	0.031 (0.66)	-0.103 (0.17)

Data are β (standardized coefficient) and p value from linear regression models adjusted for age, sex and the level of premorbid intellectual impairment with cognitive measures as outcomes.

Abbreviations: Aβ=β-amyloid; CRT=Cued Recall test; DS-MSE=Down Syndrome Mental Status Examination; GFAP=glial fibrillary acidic protein; NfL=neurofilament light chain; P-tau=phosphorylated tau; T-tau=total tau.

© 2022 Janelidze S et al. *JAMA Neurology.*

eTable 7. Associations with tau-PET in participants with DS aged ≥35y

TAU-PET<br>TEMPORAL META-ROI	MODEL PERFORMANCE		
**Univariable models**	**OR (p value)**	**AUC (95% CI)**	**AIC**
Age	1.31 (<0.001)	0.878 (0.791-0.966)	53.4
P-tau217	2.52 (<0.001)	0.933 (0.873-0.993)	50.3
GFAP	2.14 (<0.001)	0.929 (0.874-0.984)	56.6
NfL	1.24 (0.044)	0.843 (0.755-0.931)	80.1
T-tau	4.80 (0.008)	0.757 (0.627-0.887)	85.6
Aβ42/Aβ40	1.18 (0.29)	0.516 (0.344-0.688)	86.8
**Multivariable models<br>(age + plasma biomarker)**			
P-tau217	2.26 (0.003)	0.957 (0.903-1.0)	35.2
GFAP	1.74 (0.010)	0.936 (0.884-0.988)	46.4
T-tau	2.97 (0.09)	0.899 (0.812-0.987)	50.5
Aβ42/Aβ40	1.34 (0.11)	0.900 (0.819-0.982)	52.7
NfL	1.02 (0.83)	0.880 (0.795-0.965)	55.2
**TAU-PET<br>NEOCORTICAL META-ROI**			
**Univariable models**	**OR (p value)**	**AUC (95% CI)**	**AIC**
Age	1.33 (0.001)	0.889 (0.795-0.982)	43.9
P-tau217	2.97 (<0.001)	0.969 (0.926-1.0)	26.6
GFAP	1.96 (<0.001)	0.914 (0.846-0.982)	42.7
NfL	1.19 (0.08)	0.826 (0.724-0.928)	61.2
T-tau	1.87 (0.09)	0.809 (0.700-0.917)	60.9
Aβ42/Aβ40	1.26 (0.17)	0.539 (0.320-0.757)	62.5
**Multivariable models<br>(age + plasma biomarker)**			
P-tau217	2.46 (0.003)	0.983 (0.957-1.0)	24.5
GFAP	1.59 (0.028)	0.923 (0.849-0.997)	39.7
Aβ42/Aβ40	1.51 (0.051)	0.917 (0.836-0.999)	41.9
T-tau	1.26 (0.51)	0.897 (0.813-0.982)	45.5
NfL	1.00 (0.99)	0.890 (0.800-0.980)	45.9

Data are from logistic regression models and ROC curve analysis with tau-PET status as outcome. For plasma biomarkers, odds ratios represent increased risk of tau-PET positivity for each SD change in biomarker value.

Abbreviations: Aβ=β-amyloid; AIC=Akaike information criteria; AUC= area under the curve; CI=confidence interval; GFAP=glial fibrillary acidic protein; NfL=neurofilament light chain; OR=odds ratio; PET=positron emission tomography; P-tau=phosphorylated tau; ROC= receiver operating characteristic curve; ROI=region of interest; T-tau=total tau.

© 2022 Janelidze S et al. *JAMA Neurology.*

eTable 8. Associations with Aβ-PET in participants with DS aged ≥35y

**Biomarkers**	**Aβ-PET**		
**Univariable models**	**OR (p value)**	**AUC (95% CI)**	**AIC**
Age	1.22 (<0.001)	0.824 (0.762-0.885)	171.4
P-tau217	2.44 (<0.001)	0.909 (0.865-0.953)	122.6
GFAP	2.02 (<0.001)	0.872 (0.818-0.925)	145.5
NfL	1.48 (<0.001)	0.802 (0.733-0.871)	200.3
T-tau	3.69 (<0.001)	0.703 (0.624-0.782)	204.4
Aβ42/Aβ40	0.95 (0.44)	0.605 (0.518-0.693)	229.3
**Multivariable models (age + plasma biomarker)**			
P-tau217	2.40 (<0.001)	0.931 (0.892-0.970)	106.0
GFAP	1.72 (<0.001)	0.886 (0.837-0.934)	141.1
T-tau	4.68 (<0.001)	0.871 (0.817-0.924)	149.9
NfL	1.17 (0.055)	0.837 (0.776-0.898)	168.9
Aβ42/Aβ40	0.92 (0.24)	0.829 (0.768-0.890)	172.0

Data are from logistic regression models and ROC curve analysis with Aβ-PET status as outcome. For plasma biomarkers, odds ratios represent increased risk of Aβ-PET positivity for each SD change in biomarker value.

Abbreviations: Aβ=β-amyloid; AIC= Akaike information criteria; AUC= area under the curve; CI=confidence interval; GFAP=glial fibrillary acidic protein; NfL=neurofilament light chain; OR=odds ratio; PET=positron emission tomography; P-tau=phosphorylated tau; ROC= receiver operating characteristic curve; ROI=region of interest; T-tau=total tau

eTable 9. Associations with cognition in participants with DS aged ≥35y

**Biomarker**	**DS-MSE**	**CRT**
**Models including individual plasma biomarkers**	**β (p value), N**	**β (p value), N**
P-tau217	-0.331 (<0.001), 244	-0.528, (<0.001), 228
GFAP	-0.272 (<0.001), 244	-0.415 (<0.001), 228
T-tau	-0.130 (0.013), 211	-0.283 (<0.001), 198
NfL	-0.153 (0.018), 211	-0.376 (<0.001), 198
Aβ42/Aβ40	-0.020 (0.70), 211	-0.056 (0.38), 198
**Multivariable models combining plasma P-tau217, GFAP, NfL, T-tau**		
P-tau217	-0.271 (<0.001)	-0.457 (<0.001)
GFAP	-0.012 (0.89)	0.055 (0.57)
T-tau	-0.065 (0.22)	-0.116 (0.048)
NfL	0.024 (0.74)	-0.103 (0.18)

Data are β (standardized coefficient) and p value from linear regression models adjusted for age, sex and the level of premorbid intellectual impairment with cognitive measures as outcomes.

Abbreviations: Aβ=β-amyloid; CRT=Cued Recall test; DS-MSE=Down Syndrome Mental Status Examination; GFAP=glial fibrillary acidic protein; NfL=neurofilament light chain; P-tau=phosphorylated tau; T-tau=total tau.

© 2022 Janelidze S et al. *JAMA Neurology.*

eFigure 1

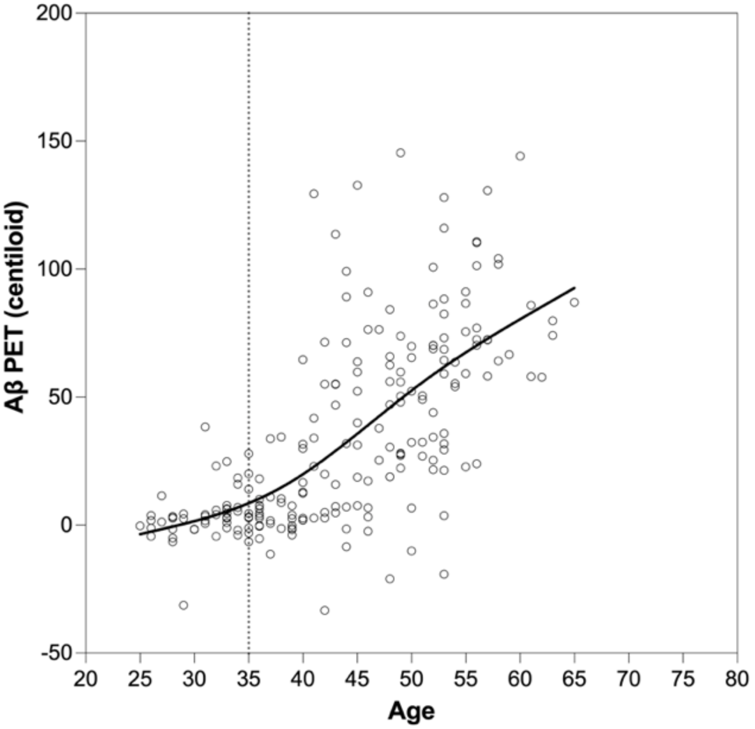

eFigure 1. Aβ-PET centiloid values as a function of age in participants with DS.

© 2022 Janelidze S et al. *JAMA Neurology.*

eFigure 2

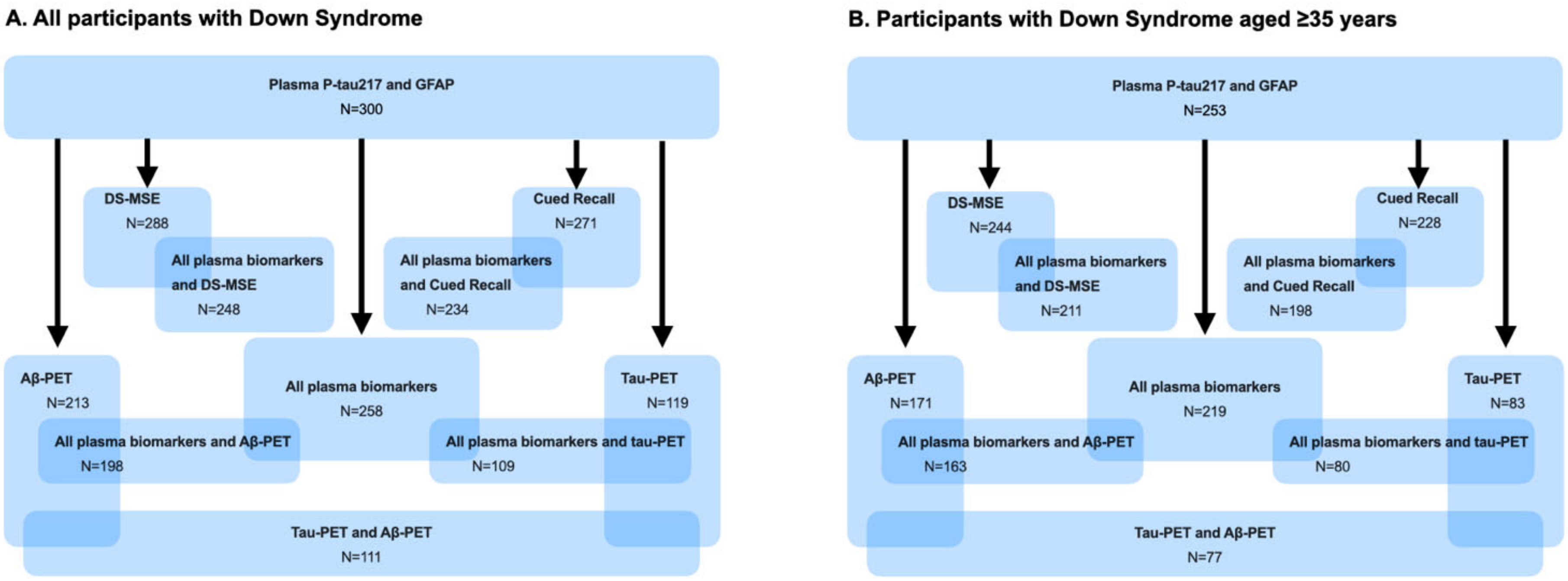

eFigure 2. The numbers of DS participants with plasma biomarker, PET and cognitive measures available included in the main (all participants, A) and sensitivity analyses (aged ≥35 years, B).

© 2022 Janelidze S et al. *JAMA Neurology.*

eFigure 3

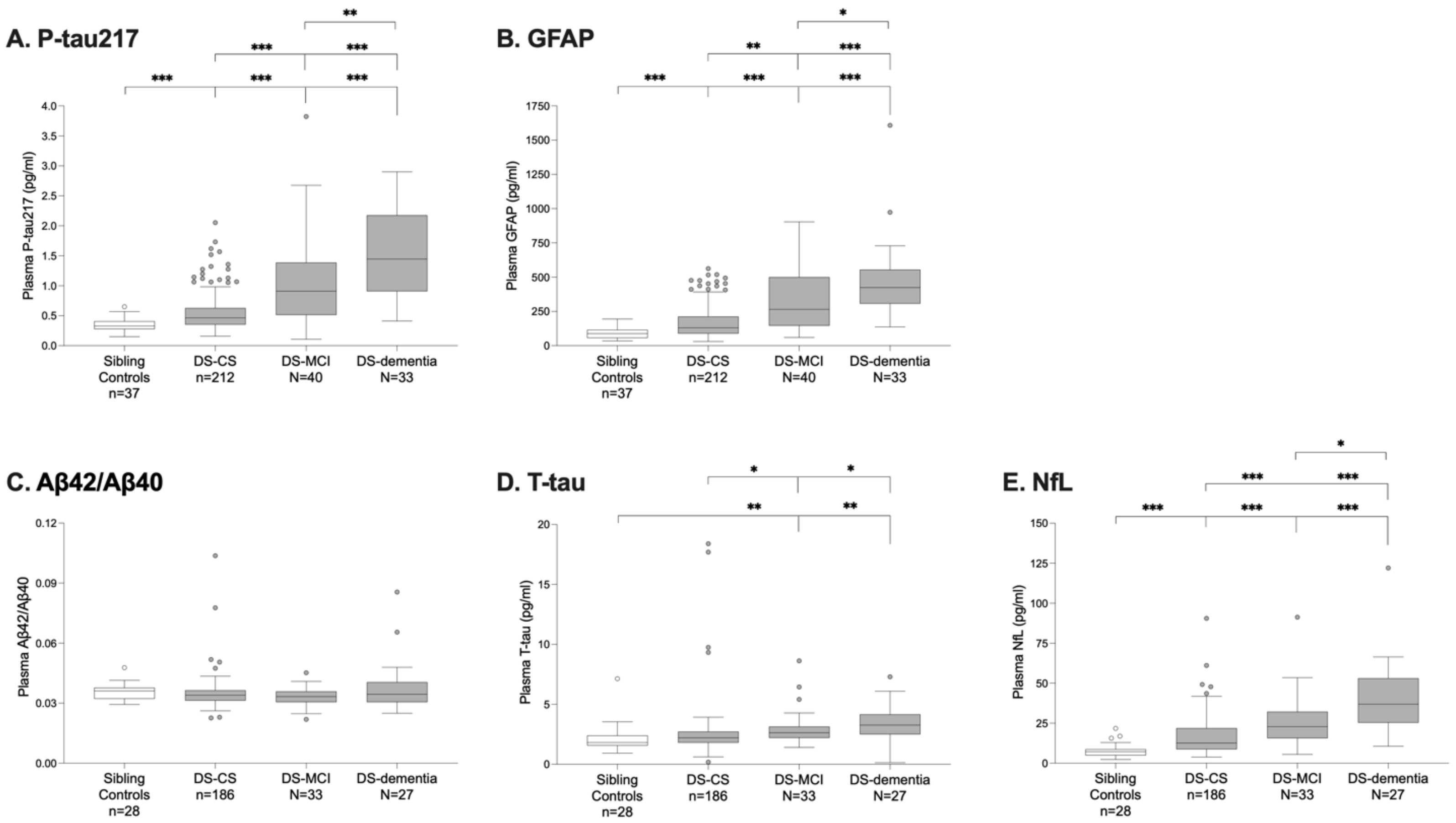

eFigure 3. Plasma biomarker concentrations across diagnostic groups. Plasma concentrations of P-tau217 (A), GFAP (B), Aβ42/Aβ40 (C), T-tau (D) and NfL (E) were compared between non-DS sibling controls, DS-CS, DS-MCI and DS-dementia. Plasma T-tau and NfL data in overlapping sample have been reported previously.[9] P-values are from univariate general linear models adjusted for age and sex with Bonferroni correction for multiple comparisons; * $p<0.05$; ** $p<0.01$; *** $p<0.001$

Abbreviations: Aβ=β-amyloid; CS=cognitively stable; DS=Down syndrome; GFAP=glial fibrillary acidic protein; MCI=mild cognitive impairment; NfL=neurofilament light chain; P-tau=phosphorylated tau; T-tau=total tau

© 2022 Janelidze S et al. *JAMA Neurology.*

eFigure 4

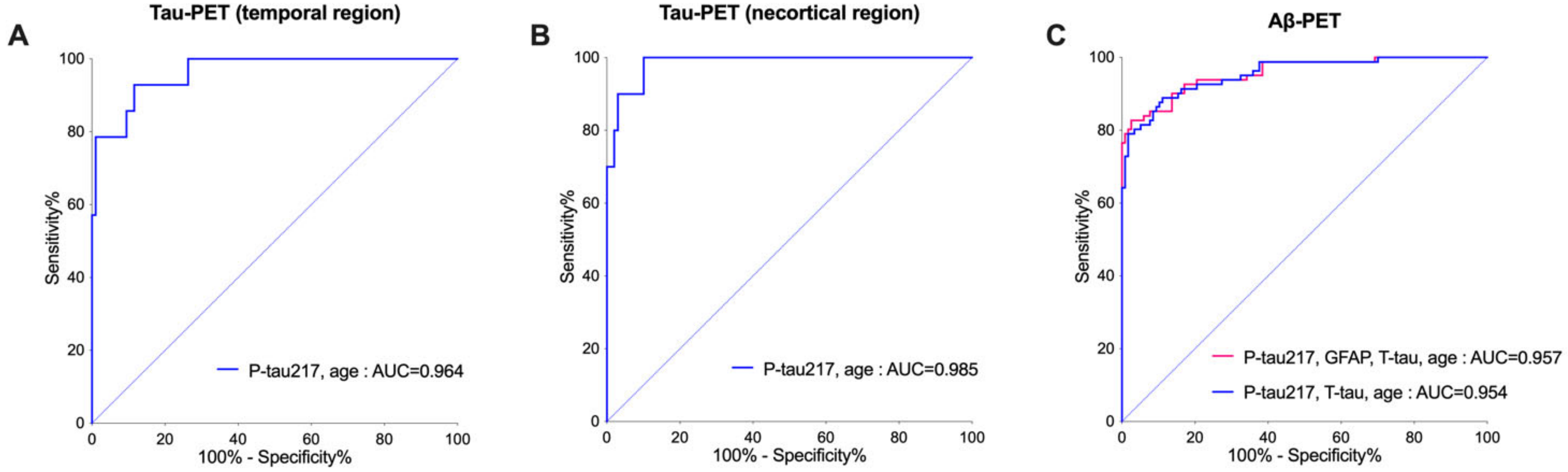

eFigure 4. ROC curve analyses for predicting abnormal Tau-PET status and Aβ-PET in participant with DS. ROC curves are shown for the best performing model which was the most parsimonious one including only P-tau217 and age as predictors to discriminate abnormal vs normal tau-PET status in the temporal and neocortical meta-ROI (A and B) and for the best performing and the most parsimonious models to discriminate abnormal vs normal Aβ-PET status (C). A, N=109 (14 tau-PET positive); B, N=109 (10 tau-PET positive); C, N=198 (81 Aβ-PET positive).

Abbreviations: Aβ=β-amyloid; DS=Down syndrome; PET=positron emission tomography; P-tau=phosphorylated tau; ROI=region of interest.

© 2022 Janelidze S et al. *JAMA Neurology.*

eFigure 5

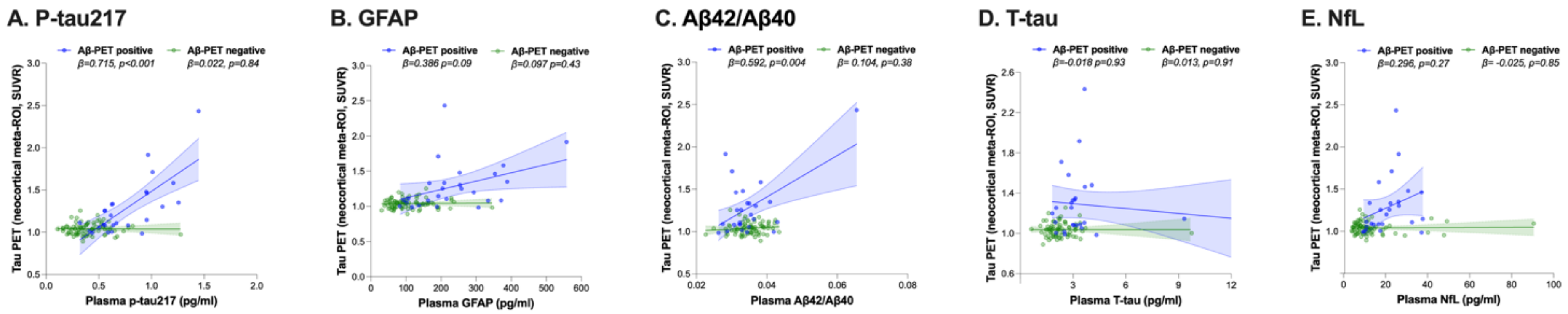

eFigure 5. Associations between plasma biomarkers and tau-PET SUVR in the neocortical region in participant with DS. Associations of plasma P-tau217 (A), GFAP (B), Aβ42/Aβ40 (C), T-tau (D) and NfL (E) with tau-PET SUVR in the neocortical meta-ROI. Data are shown as β (standardized coefficient) and p value from linear regression models adjusted for age and sex. A and B, N=111 (26 Aβ-PET positive); C-E, N=102 (26 Aβ-PET positive). One T-tau value is not shown in D but was included in the statistical analysis.

Abbreviations: Aβ=β-amyloid; DS=Down syndrome; GFAP=glial fibrillary acidic protein; NfL=neurofilament light chain; P-tau=phosphorylated tau; PET=positron emission tomography; ROI=region of interest; SUVR=standardized uptake value ratio; T-tau=total tau.

© 2022 Janelidze S et al. *JAMA Neurology.*

eFigure 6

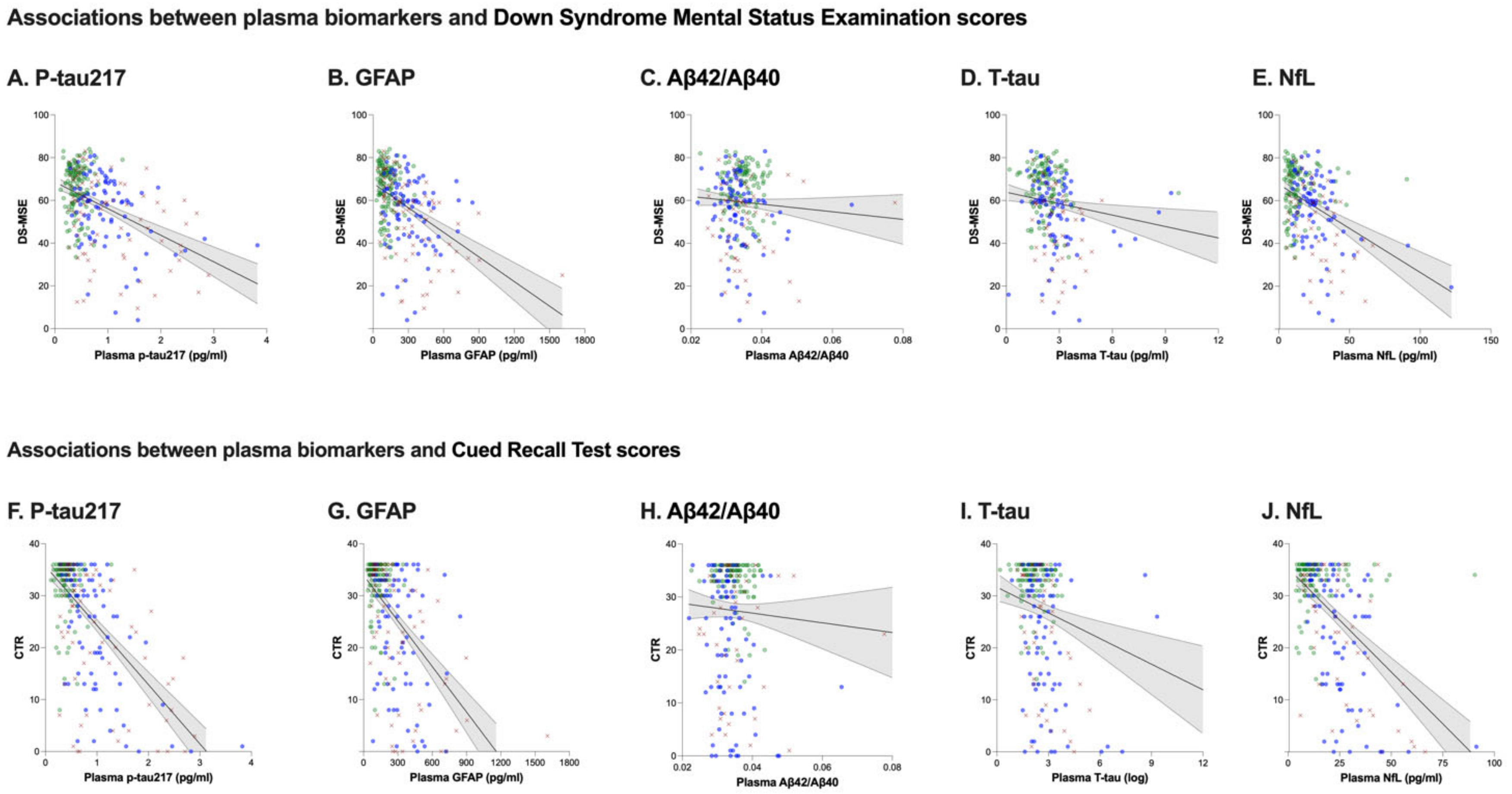

eFigure 6. Associations of plasma biomarkers with cognition in participant with DS. Associations of plasma P-tau217 (A, F), GFAP (B, G), Aβ42/Aβ40 (C, H), T-tau (D, I) and NfL (E, J) with DS-MSE (A-E) and CRT (F-J). Blue and green circles represent Aβ+ and Aβ–participants; participants with undefined Aβ status are represented as red x points. A and B, N=288; C-E, N=248; F and G, N=271; I-K, N=234. Two T-tau and three Aβ42/Aβ40 values are not shown in C, D, H and I but these data were included in the statistical analysis.

Abbreviations: Aβ=β-amyloid; DS=Down syndrome; DS-MSE=Down Syndrome Mental Status Examination; CRT=Cued Recall Test; GFAP=glial fibrillary acidic protein; NfL=neurofilament light chain; P-tau=phosphorylated tau; T-tau=total tau.

© 2022 Janelidze S et al. *JAMA Neurology.*

eReferences

1. Handen BL, Lott IT, Christian BT, et al. The Alzheimer's Biomarker Consortium-Down Syndrome: Rationale and methodology. *Alzheimers Dement (Amst).* 2020;12(1):e12065.
2. Haxby JV. Neuropsychological evaluation of adults with Down's syndrome: patterns of selective impairment in non-demented old adults. *J Ment Defic Res.* 1989;33 (Pt 3):193-210.
3. Devenny DA, Zimmerli EJ, Kittler P, Krinsky-McHale SJ. Cued recall in early-stage dementia in adults with Down's syndrome. *J Intellect Disabil Res.* 2002;46(Pt 6):472-483.
4. Hartley SL, Handen BL, Devenny D, et al. Cognitive indicators of transition to preclinical and prodromal stages of Alzheimer's disease in Down syndrome. *Alzheimers Dement (Amst).* 2020;12(1):e12096.
5. Krinsky-McHale SJ, Zigman WB, Lee JH, et al. Promising outcome measures of early Alzheimer's dementia in adults with Down syndrome. *Alzheimers Dement (Amst).* 2020;12(1):e12044.
6. Silverman W, Krinsky-McHale SJ, Zigman WB, Schupf N, and the New York Aging Research P. Adults with Down syndrome in randomized clinical trials targeting prevention of Alzheimer's disease. *Alzheimers Dement.* 2021.
7. Janelidze S, Palmqvist S, Leuzy A, et al. Detecting amyloid positivity in early Alzheimer's disease using combinations of plasma Abeta42/Abeta40 and p-tau. *Alzheimers Dement.* 2021.
8. Palmqvist S, Janelidze S, Quiroz YT, et al. Discriminative Accuracy of Plasma Phospho-tau217 for Alzheimer Disease vs Other Neurodegenerative Disorders. *JAMA.* 2020.
9. Petersen ME, Rafii MS, Zhang F, et al. Plasma Total-Tau and Neurofilament Light Chain as Diagnostic Biomarkers of Alzheimer's Disease Dementia and Mild Cognitive Impairment in Adults with Down Syndrome. *J Alzheimers Dis.* 2021;79(2):671-681.
10. Zammit MD, Tudorascu DL, Laymon CM, et al. Neurofibrillary tau depositions emerge with subthreshold cerebral beta-amyloidosis in down syndrome. *Neuroimage Clin.* 2021;31:102740.
11. Zammit MD, Tudorascu DL, Laymon CM, et al. PET measurement of longitudinal amyloid load identifies the earliest stages of amyloid-beta accumulation during Alzheimer's disease progression in Down syndrome. *Neuroimage.* 2021;228:117728.
12. Cho H, Choi JY, Hwang MS, et al. In vivo cortical spreading pattern of tau and amyloid in the Alzheimer disease spectrum. *Ann Neurol.* 2016;80(2):247-258.
13. Jack CR, Jr., Wiste HJ, Weigand SD, et al. Age-specific and sex-specific prevalence of cerebral beta-amyloidosis, tauopathy, and neurodegeneration in cognitively unimpaired individuals aged 50-95 years: a cross-sectional study. *Lancet Neurol.* 2017;16(6):435-444.
14. Klunk WE, Koeppe RA, Price JC, et al. The Centiloid Project: standardizing quantitative amyloid plaque estimation by PET. *Alzheimers Dement.* 2015;11(1):1-15 e11-14.

© 2022 Janelidze S et al. *JAMA Neurology.*

eTable 10. Members of the Alzheimer's Biomarker Consortium - Down Syndrome (ABC-DS)					
***First Name and Middle Initial(s)**	***Last Name**	Academic Degrees	Institution	Location	Role or Contribution
Howard J.	Aizenstein	MD PhD			Co-Investigator
Beau M.	Ances	MD PhD	Washington University	St. Louis, MO USA	Co-Investigator
Howard F.	Andrews	PhD	Columbia University	New York, NY USA	Co-Investigator
Karen	Bell	MD	Columbia University	New York, NY USA	Co-Investigator
Rasmus M.	Birn	PhD	University of Wisconsin	Madison, WI USA	Co-Investigator
Adam M.	Brickman	PhD	Columbia University	New York, NY USA	Co-Investigator
Peter	Bulova	MD			Co-Investigator
Amrita	Cheema	PhD	Georgetown University	Washington, DC USA	Co-Investigator
Kewei	Chen	PhD			Co-Investigator
Bradley T.	Christian	PhD	University of Wisconsin	Madison, WI USA	Principle Investigator
Isabel	Clare	PhD	Cambridge University	Cambridge, England	Co-Investigator
Lorraine	Clark	PhD			Co-Investigator
Ann D.	Cohen	PhD	Univeristy of Pittsburgh	Pittsburgh, PA USA	Co-Investigator
John N.	Constantino	MD	Washington University	St. Louis, MO USA	Co-Investigator
Eric W.	Doran	MS	University of California, Irvine	Irvine, CA USA	Co-Investigator
Anne	Fagan	PhD	Washington University	St. Louis, MO USA	Co-Investigator
Eleanor	Feingold	PhD	University of Pittsburgh	Pittsburgh, PA USA	Co-Investigator
Tatiana M.	Foroud	PhD	National Centralized Repository for Alzheimer's Disease and Related Dementias	Indianapolis IN USA	Co-Investigator
Benjamin L.	Handen	PhD	Univeristy of Pittsburgh	Pittsburgh, PA USA	Principle Investigator
Sigan L.	Hartley	PhD	University of Wisconsin	Madison, WI USA	Co-Investigator
Elizabeth	Head	PhD	University of California, Irvine	Irvine, CA USA	Co-Investigator
Rachel	Henson	PhD	Washington University	St. Louis, MO USA	Co-Investigator
Christy	Hom	PhD	University of California, Irvine	Irvine, CA USA	Co-Investigator
Lawrence	Honig	MD	Columbia University	New York, NY USA	Co-Investigator

© 2022 Janelidze S et al. *JAMA Neurology.*

Milos D.	Ikonomovic	MD	Univeristy of Pittsburgh	Pittsburgh, PA USA	Co-Investigator
Sterling C.	Johnson	PhD	University of Wisconsin	Madison, WI USA	Co-Investigator
Courtney	Jordan	RN	Harvard Medical School	Boston, MA USA	Co-Investigator
M. Ilyas	Kamboh	PhD	University of Pittsburgh	Pittsburgh, PA USA	Co-Investigator
David	Keator	PhD	University of California, Irvine	Irvine, CA USA	Co-Investigator
William E.	Klunk	MD PhD	Univeristy of Pittsburgh	Pittsburgh, PA USA	Principle Investigator
Julia K.	Kofler	MD	Univeristy of Pittsburgh	Pittsburgh, PA USA	Co-Investigator
William Charles	Kreisl	MD	Univeristy of Pittsburgh	Pittsburgh, PA USA	Co-Investigator
Sharon J.	Krinsky-McHale	PhD	New York Institute for Basic Research in Developmental Disabilities	New York, NY USA	Co-Investigator
Florence	Lai	MD	Harvard Medical School	Boston, MA USA	Co-Investigator
Patrick	Lao	PhD	University of Wisconsin	Madison, WI USA	Co-Investigator
Charles	Laymon	PhD	Univeristy of Pittsburgh	Pittsburgh, PA USA	Co-Investigator
Joseph H.	Lee	PhD	Columbia University	New York, NY USA	Co-Investigator
Ira T.	Lott	MD	University of California, Irvine	Irvine, CA USA	Principle Investigator
Victoria	Lupson	PhD			Co-Investigator
Mark	Mapstone	PhD	University of California, Irvine	Irvine, CA USA	Co-Investigator
Chester A.	Mathis	PhD	Univeristy of Pittsburgh	Pittsburgh, PA USA	Co-Investigator
Davneet Singh	Minhas	PhD			Co-Investigator
Neelesh	Nadkarni	MD			Co-Investigator
Sid	O-Bryant	PhD	University of North Texas Health Sciences	Ft. Worth, TX USA	Co-Investigator
Deborah	Pang	MPH	Columbia University	New York, NY USA	Co-Investigator
Melissa	Petersen	PhD	University of North Texas Health Sciences	Ft. Worth, TX USA	Co-Investigator
Julie C.	Price	PhD	Harvard Medical School	Boston, MA USA	Co-Investigator
Margaret	Pulsifer	PhD	Harvard Medical School	Boston, MA USA	Co-Investigator
Michael	Rafii	PhD	University of Southern California	Los Angeles, CA USA	Co-Investigator
Eric	Reiman	MD			Co-Investigator
Batool	Rizvi	MS	University of Wisconsin	Madison, WI USA	Co-Investigator

© 2022 Janelidze S et al. *JAMA Neurology.*

Herminia Diana	Rosas	MD	Harvard Medical School	Boston, MA USA	Co-Investigator
Nicole	Schupf	PhD	Columbia University	New York, NY USA	Principle Investigator
Wayne P.	Silverman	PhD	University of California, Irvine	Irvine, CA USA	Principle Investigator
Dana L.	Tudorascu	PhD	Univeristy of Pittsburgh	Pittsburgh, PA USA	Co-Investigator
Rameshwari	Tumuluru	MD			Co-Investigator
Benjamin	Tycko	MD PhD	Hackensack Meridian Health	Nutley, NJ USA	Co-Investigator
Badri	Varadarajan	PhD			Co-Investigator
Desiree A.	White	PhD	Washington University	St. Louis, MO USA	Co-Investigator
Michael A.	Yassa	PhD	University of California, Irvine	Irvine, CA USA	Co-Investigator
Shahid	Zaman	MD PhD	Cambridge University	Cambridge, England	Co-Investigator
Fan	Zhang	PhD	University of North Texas Health Sciences	Ft. Worth, TX USA	Co-Investigator

© 2022 Janelidze S et al. *JAMA Neurology.*
